# Quantum biology: From mechanisms to medicine

**DOI:** 10.1002/ctm2.70694

**Published:** 2026-05-18

**Authors:** Ji‐Yong Sung, Jae‐Ho Cheong

**Affiliations:** ^1^ Department of Neurosurgery Seoul National University Bundang Hospital Seoul National University College of Medicine Seongnam‐si Republic of Korea; ^2^ Institute for Convergence Research and Education in Advanced Technology Yonsei University Seoul Republic of Korea; ^3^ Department of Quantum Information Graduate School Yonsei University Incheon Republic of Korea; ^4^ Department of Surgery Yonsei University College of Medicine Seoul Republic of Korea

**Keywords:** DNA proton tautomerisation and mutation, mitochondrial electron transport and ROS, quantum biology, quantum coherence and tunnelling, quantum‐informed medicine, radical pair mechanism, spin dynamics

## Abstract

**Background:**

Quantum biology explores how quantum mechanical phenomena—including coherence, tunneling, superposition, and spin dynamics—contribute to biological function. Although once considered negligible in warm and noisy biological environments, increasing evidence suggests that quantum effects play important roles in diverse living systems.

**Objective:**

This review aims to summarize the current understanding of quantum biological mechanisms, highlight their relevance to physiology and disease, and discuss emerging biomedical and technological applications.

**Methods:**

We reviewed recent experimental, computational, and theoretical advances in quantum biology, including studies employing ultrafast spectroscopy, quantum sensing, cryo‐electron microscopy, and quantum simulation approaches. Key biological systems examined include photosynthetic complexes, enzymatic reactions, DNA base pairing, sensory systems, and mitochondrial electron transport.

**Results:**

Accumulating evidence indicates that quantum coherence, tunneling, and spin‐dependent processes contribute to photosynthetic energy transfer, enzymatic catalysis, proton transfer in DNA, magnetoreception, olfaction, and mitochondrial bioenergetics. Advances in quantum sensing and computational modeling have further enabled direct investigation of coherence dynamics and electron transfer mechanisms in biological systems. These findings suggest that quantum effects may influence aging, cancer, neurodegeneration, and metabolic dysfunction through mechanisms involving reactive oxygen species production, mutagenesis, and altered redox signaling.

**Conclusion:**

Quantum biology is evolving from a speculative concept into an experimentally accessible and translationally relevant discipline. Integrating quantum principles with systems biology, multi‐omics, and precision medicine may provide new opportunities for diagnostics, biomarker discovery, and therapeutic development. Continued advances in spectroscopy, quantum sensing, and quantum computing are expected to further establish the role of quantum phenomena in health and disease.

## Key points

- Quantum biology reveals that coherence, tunnelling and spin dynamics are not rare anomalies but fundamental design principles that optimise biological efficiency, precision and adaptability across living systems.
- Recent advances in ultrafast spectroscopy, quantum sensing, cryo‐EM and quantum computing have transformed quantum biology from a speculative theory into a data‐driven, experimentally testable science.
- Quantum phenomena underlie key biological processes – from photosynthetic energy transfer and DNA mutagenesis to mitochondrial respiration, sensory perception and cognition – offering new mechanistic insights into health and disease.
- Understanding decoherence, tunnelling and entanglement in biological contexts opens new frontiers for quantum‐informed diagnostics, biomarkers and therapeutics, with direct implications for cancer, aging and neurodegenerative diseases.
- The convergence of quantum mechanics and biology suggests a future of precision medicine guided not only by genetic and molecular data but also by the quantum state of biomolecular processes.

## Graphical abstract

*Quantum biology across systems: from mechanisms to medicine*. This graphical abstract highlights how quantum phenomena‐coherence, tunnelling and spin dynamics‐shape fundamental biological processes and point towards quantum‐informed medicine. In photosynthesis, quantum coherence supports near‐lossless exciton transport across pigment–protein complexes, enabling highly efficient energy conversion. In DNA, proton tunnelling through hydrogen bonds can give rise to short‐lived tautomeric configurations, which elevate the likelihood of mispairing during replication and thereby contribute to spontaneous mutations at oncogenic hotspots. In mitochondria, quantum decoherence disrupts electron transport chain redox coupling, elevating ROS production and accelerating aging, metabolic dysfunction and cancer progression. In the brain, tunnelling and coherence influence ion dynamics in neuronal channels, potentially contributing to cognition, consciousness and rapid signal transduction. In sensory systems, quantum mechanisms underlie magnetoreception through spin‐correlated radical pairs in cryptochromes, olfactory discrimination through vibrationally assisted tunnelling and visual phototransduction through highly efficient retinal isomerisation. At the centre, these discoveries converge towards quantum‐informed medicine, which envisions diagnostics such as coherence lifetime sensors and tunnelling‐based mutational signatures, and therapeutics including mitochondrial redox modulators and DNA‐stabilising agents that directly target the quantum foundations of health and disease.

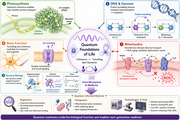

## 1 INTRODUCTION

For decades, biology has been interpreted largely through a classical lens. Cells have been described as ensembles of biochemical reactions governed by thermodynamics and kinetics, where diffusion, collision theory and energy barriers dictate rates and outcomes. Within this framework, living systems are modelled as warm, wet and noisy environments, where stochastic chemical processes dominate. This perspective has provided remarkable success in explaining enzyme catalysis, metabolic pathways and molecular signalling. However, a growing body of evidence suggests that living systems may exploit quantum mechanical resources – such as coherence, tunnelling, spin dynamics and entanglement – in ways that directly influence function,^1^, ^2^, ^3^, ^4^, ^5^ Phenomena once considered negligible in biology now appear to shape fundamental processes: long‐lived coherence in photosynthetic complexes, hydrogen and electron tunnelling in enzymatic catalysis, spin‐correlated radical pairs in magnetoreception. Odour perception can involve vibrational mechanisms, while in DNA, proton tunnelling may trigger tautomeric shifts within base pairs. These examples indicate that quantum effects, rather than being fully suppressed by thermal noise, may be stabilised or even enhanced by environmental interactions – a concept captured in theories of environment‐assisted quantum transport (ENAQT). Thus, quantum biology calls for a reframing of biological explanations, shifting from a strictly classical paradigm towards one that recognises quantum contributions as functionally significant.^3^, ^6^, ^7^

### 1.1 Classical biology versus quantum biology

The distinction between classical and quantum approaches lies in the scale, descriptors and design principles used to explain biological function. Classical biology relies on entropic mixing, diffusion‐driven encounters and barrier‐lowering catalysis. Reaction rates are modelled as functions of activation energy and temperature.^8^

Quantum biology, in contrast, emphasises wavefunction phase relationships, discrete energy levels, tunnelling probabilities and spin correlations. Biological systems are described as open quantum systems, where coherence may persist on femtosecond‐to‐picosecond timescales, often shaped by coupling to vibrational modes of the molecular environment. This leads to different design principles: while classical optimisation depends on lowering barriers or increasing reactant concentrations, quantum optimisation may involve energy‐level matching, vibrational resonance or coupling strengths that stabilise coherence or promote tunnelling. For example, pigment–protein complexes in photosynthesis^9^, ^10^ appear to harness vibrational environments to guide excitonic transfer,^11^ while mitochondrial electron transport may depend on the delicate balance of electronic coupling among redox centres.

Similarly, mitochondrial electron transport proceeds through a chain of redox‐active centres, where the efficiency and directionality of electron transfer are governed by distance‐dependent electronic coupling and quantum mechanical tunnelling between these sites.^12^, ^13^, ^14^, ^15^

These ideas have become experimentally accessible. Two‐dimensional electronic spectroscopy (2DES) probes electronic and excited‐state dynamics, including charge transfer processes, and can provide indirect insight into redox‐dependent changes in biomolecular systems. Femtosecond ultrafast spectroscopy uncovers coherent oscillatory dynamics, while diamond nitrogen–vacancy (NV) centres enable nanoscale detection of magnetic and electric fields inside living cells, and cryo‐EM integrated with quantum simulations can model charge localisation and electron‐transfer bottlenecks with near‐atomic resolution.^3^, ^16^, ^17^, ^18^, ^19^

### 1.2 Historical origins: From Schrödinger to photosynthesis

The conceptual foundations of quantum biology can be linked to Erwin Schrödinger's classic work What is Life?,^20^, ^21^ which introduced concepts such as the ‘aperiodic crystal’ and order‐from‐order as bridges between physics and biology. Later, quantum chemistry provided a framework for understanding electronic structures and chemical bonds, whereas enzymology revealed evidence of hydrogen tunnelling through kinetic isotope effect (KIE) measurements. For many years, doubts remained – was it really possible for quantum phenomena to endure and operate within biological environments overwhelmed by thermal fluctuations?^22^

The turning point came with the advent of ultrafast 2DES in the late 2000s. Experiments on photosynthetic pigment–protein complexes (e.g., the Fenna–Matthews–Olson [FMO] complex, PSI/PSII) revealed long‐lived coherence signatures at physiological temperatures.^23^, ^24^ This overturned the assumption that coherence must vanish in warm, noisy environments.^25^ Around the same time, theoretical and computational models began exploring proton tunnelling in DNA and electron tunnelling in mitochondrial Complex I, suggesting that quantum biology extended well beyond photosynthesis.^12^, ^26^, ^27^, ^28^

**FIGURE 1:**
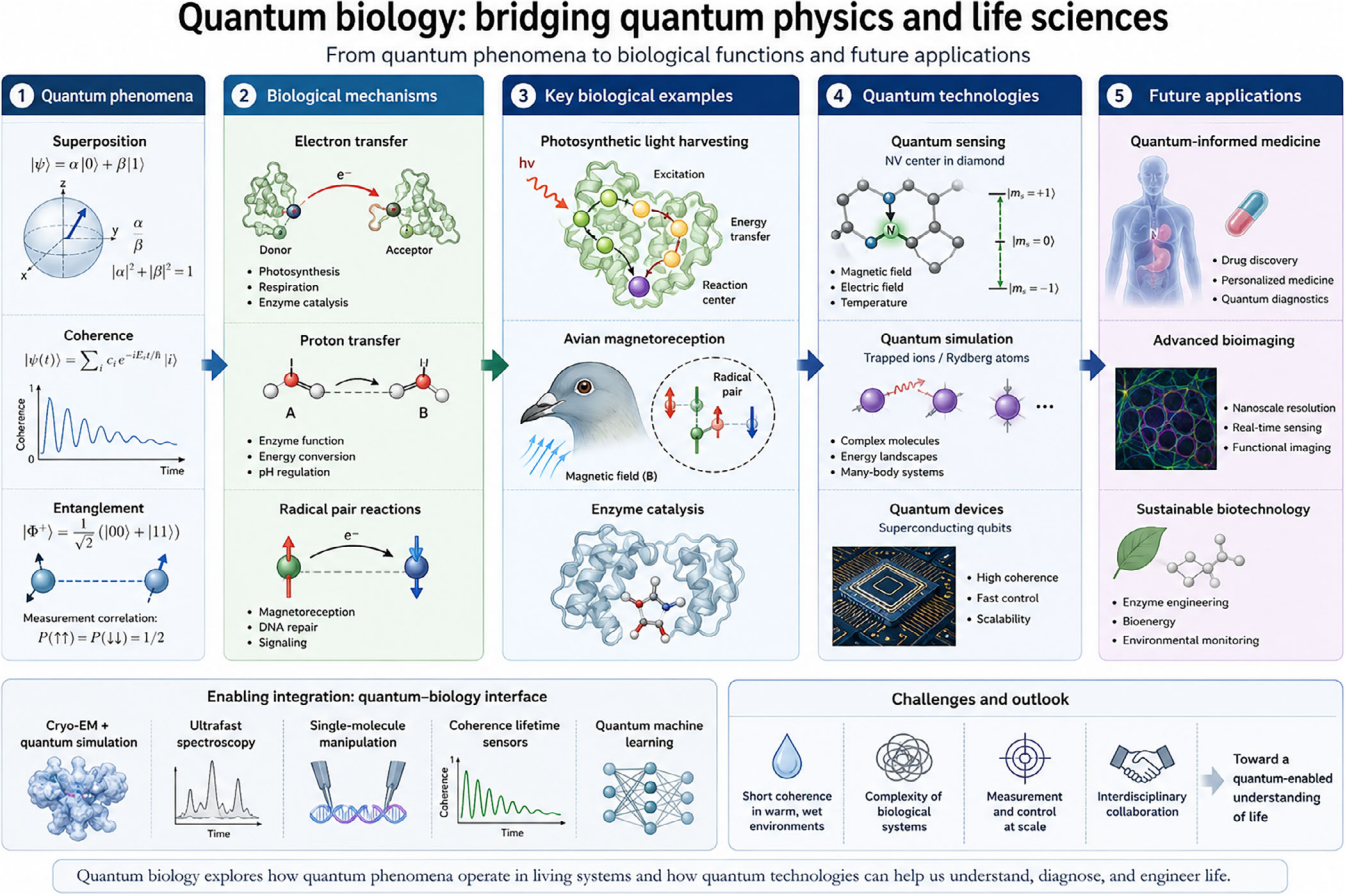
From Schrödinger's ‘What is Life?’ to quantum‐informed medicine. This conceptual diagram illustrates the intellectual and scientific trajectory of quantum biology. Beginning with Schrödinger's 1944 question ‘What is Life?’, the figure traces the translation of quantum principles into biological mechanisms and ultimately into medicine. The honeycomb crystal symbolises Schrödinger's ‘aperiodic crystal’ and the molecular basis of heredity. Waveforms and molecular orbitals represent quantum coherence, tunnelling and spin phenomena that shape biological function. These insights converge into modern quantum biology, depicted here as bridging fundamental physics with living systems. The human silhouette with highlighted organs represents translational applications in quantum‐informed medicine, where coherence lifetimes, tunnelling signatures and spin states can be leveraged for diagnostics and therapies.

More recently, applications in cancer biology have demonstrated that ultrafast spectroscopy can distinguish coherence lifetimes in healthy versus tumour mitochondria, NV sensing can map ROS flux and mitochondrial membrane potentials and cryo‐EM combined with variational quantum eigensolver (VQE) approaches can model disrupted electron delocalisation in pathogenic mutations.^29^ These advances have transformed quantum biology from speculative theory into a measurable, experimentally testable discipline (Figure 1).^30^

### 1.3 Next‐generation drivers: ultrafast spectroscopy, quantum computing and advances in biophysics

Several technological breakthroughs now drive the rapid expansion of quantum biology:

*Ultrafast spectroscopy*. 2DES resolves coherence dynamics in femtoseconds, revealing how excitonic transfer, redox states and protein vibrations interact in real time. These measurements have shown altered coherence lifetimes in diseased versus healthy systems, providing direct evidence of quantum effects in living biology.

*Quantum sensing*.^31^, ^32^ NV centres in diamond function as quantum sensors under ambient conditions, capable of detecting magnetic fields, electric potentials and ROS flux in living cells at nanoscale resolution. This enables real‐time correlation of altered quantum coherence with phenotypic outcomes such as apoptosis resistance or metabolic reprogramming.

*Cryo‐EM* ^33^ *and quantum simulation*. High‐resolution cryo‐electron microscopy (EM) provides structural maps of large biomolecular complexes. Coupled with quantum algorithms (e.g., VQE, density matrix renormalisation group [DMRG]), these structures can be analysed to reveal electron‐transfer bottlenecks, charge localisation and coherence loss in mutant proteins.

*Quantum computing and quantum machine learning (QML)*. Even in today's NISQ era, hybrid algorithms like VQE and QAOA allow simulations of multi‐electron biomolecular Hamiltonians that are intractable on classical supercomputers. By integrating quantum support vector machines (QSVMs) with hybrid neural networks, researchers can enhance the interpretation of multi‐omics profiles, protein‐folding dynamics and ligand–receptor interactions, thereby advancing both biomarker discovery and drug development.^33^

*Precision biophysics*. By integrating ultrafast spectroscopy, nanoscale quantum sensing and quantum‐classical computation, modern biophysics provides a pipeline to observe, model and engineer biological quantum effects. This workflow supports not only mechanistic understanding but also translational applications, including the design of quantum‐informed biomarkers and therapeutics.

## 2 CORE QUANTUM PHENOMENA IN BIOLOGY

The dynamics of living matter emerge from the interplay of quantum physics and biochemical principles. Biological systems are most often associated with quantum phenomena such as coherence, tunnelling and spin‐related entanglement dynamics. While these processes are well established in physics, their survival and functional significance in warm, noisy biological environments represent one of the most fascinating questions in modern science.

### 2.1 Quantum coherence

#### 2.1.1 Definition and biological relevance

Quantum coherence refers to the phase correlation between quantum states, allowing them to exist as superpositions rather than collapsing into distinct outcomes. Within living systems, quantum coherence allows excitations or charge carriers to sample many routes at once, thereby enhancing both transport efficiency and accuracy. In this review, the term ‘coherence lifetime’ denotes the characteristic timescale over which these phase relationships are preserved before decoherence due to environmental interactions. Importantly, we distinguish between electronic coherence (phase correlations between electronic states), vibrational coherence (coherent nuclear motion) and vibronic coherence (coupled electronic–vibrational states), as these forms have distinct physical origins and functional implications in biological systems.

Early studies reported long‐lived quantum coherence in photosynthetic complexes based on oscillatory signals observed in Engel et al.^11^ However, subsequent work has reinterpreted these oscillations as arising, at least in part, from vibronic (electronic–vibrational) coherence rather than purely electronic coherence. Current understanding suggests that the functional role and nature of coherence in photosynthetic energy transfer remain subjects of ongoing debate, with evidence supporting contributions from both electronic and vibrational dynamics.^18^, ^34^

#### 2.1.2 Photosynthesis as a paradigm

A well‐known case of quantum coherence in living systems is observed within photosynthetic complexes. Studies of the Fenna–Matthews–Olson (FMO) complex in green sulphur bacteria, and later in higher plant complexes such as Photosystem I (PSI) and Photosystem II (PSII), suggested that excitonic energy transfer between pigment molecules is not purely stochastic, but may include contributions from quantum coherence under specific conditions. Instead, ultrafast 2DES detected long‐lived oscillations, interpreted as signatures of electronic coherence,^11^ even at physiological temperatures. This means that excitons effectively ‘sample’ multiple pathways simultaneously before selecting the most efficient route to the reaction centre.^8^, ^25^

#### 2.1.3 Excitonic transport within chromophores

In pigment–protein assemblies, excitons are delocalised across several chromophores, creating wave‐like energy transport rather than stepwise hopping. Coupling to specific vibrational modes of the protein scaffold appears to stabilise coherence by coupling electronic and nuclear dynamics. This concept of ENAQT suggests that decoherence, rather than destroying quantum effects, can actually optimise them by mitigating localisation effects. Such findings challenge the notion that noise is purely detrimental, instead framing it as a functional component of quantum‐biological design.^35^, ^36^

Together, these studies establish coherence not as a rare anomaly, but as a robust and biologically advantageous feature of energy transfer in living systems.

### 2.2 Quantum tunnelling

#### 2.2.1 Definition and mechanisms

Quantum tunnelling occurs when a particle passes through an energy barrier higher than its classical kinetic energy would allow. In biology, tunnelling has been implicated for both electrons and protons, influencing enzymatic catalysis, respiration and proton‐transfer‐mediated tautomerisation in DNA base pairs.^37^

#### 2.2.2 Quantum tunnelling in enzymatic catalysis

A classic example is alcohol dehydrogenase, where hydride transfer exhibits KIEs too large to be explained by classical transition‐state theory. The rates align with models of electron tunnelling^38^ modulated by protein dynamics. Similarly, in the mitochondrial Complex I (NADH:ubiquinone oxidoreductase), electron transfer across flavin mononucleotide (FMN) and iron–sulphur (Fe–S) clusters spans distances of up to 14 Å, consistent with long‐range quantum‐mediated electron transfer. Here, tunnelling provides a natural explanation for efficient electron flow, linking quantum mechanics to the bioenergetic core of the cell.^12^, ^15^, ^39^

#### 2.2.3 Proton tunnelling in hydrogen‐bond networks and DNA

Protons, being lightweight particles, tunnel readily across hydrogen bonds. This has implications for proton‐transfer dynamics in DNA base pairs: base pairs can undergo transient tautomerisation (e.g., keto → enol, amino → imino) via proton tunnelling.^40^ Though fleeting, these alternative forms can mispair during replication, leading to point mutations.^26^ This mechanism provides a possible quantum‐mechanical contribution to spontaneous mutagenesis, complementing classical models of chemical instability or environmental damage. Proton tunnelling^41^ is also implicated in hydrogenbond networks in enzymes and membranes, where it can contribute to proton‐coupled electron transfer (PCET), an important process in biological energy conversion, including photosynthesis and aspects of respiration.^42^, ^43^, ^44^

#### 2.2.4 Implications for biology

Recognising the role of tunnelling has transformed how enzyme kinetics are interpreted. Instead of being limited by over‐the‐barrier activation energies, reaction rates may reflect the probability amplitudes of quantum penetration, modulated by protein conformational fluctuations.^37^ In the genetic context, tunnelling‐driven tautomerisation introduces an intrinsic quantum contribution to mutation rates and genome evolution^26^, ^45^ with consequences for adaptation, disease and aging.^45^, ^46^

### 2.3 Quantum entanglement and spin dynamics

#### 2.3.1 Entanglement in biological systems

Quantum entanglement is a quantum mechanical phenomenon characterised by non‐classical correlations between quantum states, such that the state of the system cannot be described independently of its constituent components, even when spatially separated.

In biology, entanglement is thought to manifest through spin dynamics in radical pairs—short‐lived molecular species with unpaired electrons whose spins are correlated.^47^

#### 2.3.2 Radical pair mechanism in cryptochromes

The most celebrated example is avian magnetoreception. Birds and other migratory species appear to sense the Earth's magnetic field using cryptochrome proteins in the retina. When excited by blue light, cryptochromes generate radical pairs whose spin states (singlet vs. triplet) interconvert in ways that are sensitive to weak geomagnetic fields. The ability of organisms to detect fields as weak as ∼50 µT strongly suggests reliance on quantum spin coherence and entanglement, rather than classical mechanisms.^47^, ^48^, ^49^, ^50^

#### 2.3.3 Spindependent reactions in photosynthesis and enzymatic pathways

Spin dynamics^51^ are also relevant in photosynthetic reaction centres, where radical pairs form during charge separation. The yield and directionality of electron transfer can depend on spin correlations, suggesting that quantum spin effects modulate energy conversion efficiency. Enzymes such as flavoproteins also generate radical intermediates whose reactivity may be governed by entangled spin states.

#### 2.3.4 Potential roles in signalling cascades

Although less well explored, spin‐dependent reactions could influence cellular signalling by modulating the lifetime of reactive intermediates such as reactive oxygen species (ROS).^52^, ^53^ Spin chemistry^54^ may thereby intersect with redox signalling, circadian rhythms and stress responses. The possibility that entanglement contributes to biological information processing remains speculative, but it has gained traction as evidence for spin coherence in biological conditions continues to accumulate.^17^, ^55^

## 3 QUANTUM BIOLOGY ACROSS SYSTEMS

Fundamental quantum effects—such as coherence, tunnelling and spin‐related processes—appear in many living systems, ranging from the light‐capturing complexes of photosynthetic microbes and plants to the sensory mechanisms found in complex animals. In each case, quantum effects appear not as incidental curiosities but as integral mechanisms that enhance efficiency, precision or adaptability.

### 3.1 Photosynthesis and energy transfer

#### 3.1.1 The FMO complex and light‐harvesting antennae

Photosynthesis represents perhaps the most striking example of functional quantum biology. The FMO complex^56^, ^57^ in green sulphur bacteria acts as an energy ‘wire’, transferring excitonic energy from light‐harvesting antennae to the reaction centre with remarkable efficiency. Quantum mechanical models, supported by ultrafast spectroscopy, reveal that excitons are delocalised over multiple pigments, allowing them to explore parallel pathways simultaneously.^58^

#### 3.1.2 Quantum efficiency beyond classical limits

Photosynthetic organisms routinely achieve quantum efficiencies exceeding 90%, meaning that nearly every absorbed photon is converted into charge separation at the reaction centre. Such performance is difficult to explain by purely classical hopping models, which would incur significant losses due to dissipation. Instead, coherence enables wave‐like transport, minimising the risk of exciton trapping and enhancing the probability of successful energy delivery.^59^, ^60^

#### 3.1.3 Coherence at physiological temperatures

A major breakthrough came when 2DES revealed long‐lived coherence signatures not only at cryogenic temperatures but also under near‐physiological conditions. This demonstrated that quantum coherence is not an artifact of low‐temperature physics but a robust feature of living systems, sustained by interactions with protein vibrational modes. In the ENAQT framework, environmental noise is not merely disruptive but can actively aid living systems, helping sustain quantum coherence over extended timescales and promoting more effective energy transfer.^61^, ^62^, ^63^

### 3.2 Enzymatic catalysis

#### 3.2.1 Hydrogen tunnelling in oxidoreductases

Enzymatic catalysis, traditionally explained through transition‐state stabilisation, often exhibits kinetic behaviours that defy classical models. Many oxidoreductases—including alcohol dehydrogenase, lactate dehydrogenase and soybean lipoxygenase‐show evidence of hydrogen tunnelling, where protons or hydrides cross barriers via quantum penetration rather than thermal activation.^64^, ^65^, ^66^

#### 3.2.2 Isotope substitution as a diagnostic tool

One of the strongest lines of evidence comes from isotope substitution studies. Replacing hydrogen with deuterium or tritium significantly alters reaction rates in ways that far exceed classical KIE predictions. These anomalies are well explained by tunnelling models, which account for the lighter mass and higher tunnelling probability of hydrogen.^67^, ^68^, ^69^

#### 3.2.3 Implications for biomedicine

Although still emerging, insights from quantum tunnelling in enzymatic catalysis may inform future biomedical applications, including drug design and metabolic regulation. For drug design, inhibitors that alter barrier shape or hydrogen‐bond networks may specifically disrupt tunnelling pathways. In metabolic regulation, mutations that shift protein dynamics can modulate tunnelling probabilities, leading to altered enzymatic efficiency. Quantum enzymology thus opens new opportunities for precision pharmacology and the design of mechanism‐based therapies.^70^, ^71^, ^72^

### 3.3 DNA and genetic stability

#### 3.3.1 Proton tunnelling and tautomerisation

Accurate DNA replication relies on the secure matching of complementary nucleotide bases. However, proton tunnelling across hydrogen bonds can transiently generate tautomeric forms of bases – such as imino or enol tautomers‐that mispair during replication. Though these states last only picoseconds, they are sufficient to introduce point mutations.^73^, ^74^, ^75^, ^76^

#### 3.3.2 Spontaneous mutation rates

This mechanism provides a quantum‐level explanation for spontaneous mutations, independent of external mutagens. Because of quantum mechanical proton tunnelling, DNA carries an unavoidable background level of replication errors, which in turn influences genetic diversity and evolutionary change. Recent computational studies suggest that certain oncogenic hotspots in KRAS and TP53 may be particularly susceptible to tunnelling‐induced mutations, potentially explaining why these loci recur so frequently in cancer.^40^, ^77^

#### 3.3.3 Evolutionary implications

Beyond disease, tunnelling‐driven mutations may contribute to the balance between stability and adaptability in genomes. While excessive mutagenesis threatens genomic integrity, a low level of quantum‐driven variability may enhance evolvability, providing raw material for natural selection.^40^

### 3.4 Sensory biology

#### 3.4.1 Olfaction and the vibrational theory

The sense of smell has long posed puzzles for classical lock‐and‐key models. The vibrational theory of olfaction proposes that receptors detect not only molecular shape but also vibrational frequencies, possibly through inelastic electron tunnelling.^78^, ^79^ This would explain why some structurally distinct molecules with similar vibrational spectra elicit nearly identical odours, while structurally similar compounds may smell different. Although still debated, experiments with isotopically substituted odorants lend support to this hypothesis.^78^, ^79^, ^80^, ^81^

#### 3.4.2 Vision and rhodopsin isomerisation

Visual perception begins with the cis–trans isomerisation of retinal within rhodopsin, one of the fastest and most efficient photochemical reactions known. Quantum effects contribute to the extraordinarily high quantum yield (>65%) and speed (femtoseconds) of this process. The coherent excitation of vibrational modes appears to direct retinal isomerisation along the correct pathway, ensuring rapid and reliable signal initiation.

#### 3.4.3 Magnetoreception and cryptochrome radical pairs

In the radical pair mechanism of magnetoreception, singlet radical pairs are inherently quantum mechanically entangled at their formation. However, this entanglement is more appropriately regarded as an intrinsic feature of the underlying spin‐correlated chemical process rather than a demonstrated functional driver of magnetic sensing. Current evidence indicates that magnetic field sensitivity primarily arises from spin dynamics and singlet–triplet interconversion governed by hyperfine interactions, while the direct functional role of entanglement in biological magnetoreception remains unproven.^82^, ^83^

Migratory birds and other animals can sense the Earth's magnetic field, a feat explained by the radical pair mechanism in cryptochrome proteins. When photoactivated, cryptochromes generate radical pairs whose spin states (singlet vs. triplet) are sensitive to weak geomagnetic fields. Spin coherence and entanglement allow organisms to perceive fields as weak as 50 µT, well below classical detection thresholds. This provides one of the most compelling examples of functional quantum entanglement in biology.^84^, ^85^, ^86^, ^87^

### 3.5 Mitochondrial bioenergetics

#### 3.5.1 Electron tunnelling in the respiratory chain

Mitochondria house the electron transport chain (ETC), where redox centres such as FMN and Fe–S clusters in Complex I mediate electron transfer over nanometer distances. Classical diffusion models cannot account for such efficiency; instead, electron tunnelling provides a natural explanation. Coupling between redox sites ensures rapid electron flow necessary for ATP production.^88^

#### 3.5.2 Quantum coherence in electron transfer

Emerging evidence suggests that coherence may play a role in synchronising electron motion across multiple redox centres, thereby reducing energy losses and optimising mitochondrial bioenergetics. In this context, ‘mitochondrial coherence’ refers to the transient preservation of phase relationships during electron transfer among redox centres within the ETC, such as FMN and Fe–S clusters, distinguishing it from excitonic coherence observed in photosynthetic systems. This coherence is characterised by a finite coherence lifetime, defined as the timescale over which these phase relationships are maintained before decoherence occurs. Ultrafast spectroscopic studies have revealed that coherence lifetimes differ between healthy and cancerous mitochondria, linking quantum electron transfer dynamics to disease‐associated bioenergetic dysfunction.

#### 3.5.3 ROS regulation and aging

ROS arise when electrons leak from the system due to impaired tunnelling or loss of coherent transfer.^89^, ^90^ While ROS serve as signalling molecules at low levels, their accumulation causes oxidative damage, contributing to aging and age‐related diseases. This positions quantum mitochondrial dysfunction as a central factor not only in cancer but also in broader processes of senescence and degenerative disease.^91^, ^92^

Across photosynthesis, enzymatic catalysis, genetic fidelity, sensory systems and mitochondrial metabolism, quantum mechanics emerges as a fundamental design principle of life. Coherence enables near‐perfect energy transfer, tunnelling accelerates reactions and introduces genetic variability, and entanglement through spin dynamics grants organisms extraordinary sensory capabilities. These diverse manifestations underscore the view that life does not merely tolerate quantum effects but harnesses them for functional advantage.^93^, ^94^

### 3.6 Quantum‐associated target molecules: From mechanism to disease

To move beyond system‐level descriptions, we define a set of quantum‐associated target molecules that function as convergence points linking quantum mechanisms to disease phenotypes. These molecules are selected based on three criteria: (i) the presence of experimentally or theoretically supported quantum effects, (ii) a well‐defined molecular architecture enabling mechanistic interpretation and (iii) direct relevance to disease‐associated biological processes.

Rather than reiterating individual processes, this section integrates previously described phenomena into a molecule‐centric and mechanism‐oriented framework, emphasising why these systems are particularly suitable for studying quantum biological function and how their effects can be interpreted in physiological and pathological contexts.

#### 3.6.1 Mitochondrial Complex I (FMN–Fe–S axis)

Mitochondrial Complex I is selected as a representative target because it combines a structurally defined electron transfer chain with well‐established links to disease. As outlined in Sections 3.5 and 5.1, electron transport across FMN and Fe–S clusters operates under regimes consistent with quantum tunnelling and partially coherent charge transfer. These properties can be experimentally probed using ultrafast spectroscopy and quantum sensing approaches that quantify coherence lifetimes and redox dynamics.

Functionally, these redox centres form a unified quantum functional axis, where electronic coupling and environmental interactions regulate energy transfer efficiency. Perturbations arising from mutations, oxidative stress or structural disorder lead to coherence loss and electron leakage, resulting in excessive ROS production. This establishes a direct mechanistic pathway linking quantum‐level instability to metabolic dysfunction, aging and cancer, positioning Complex I as a primary site of quantum vulnerability in disease.

#### 3.6.2 DNA base pairs as quantum mutational units

DNA base pairs are selected as minimal and ubiquitous molecular systems in which quantum effects can directly influence genetic outcomes. Building on Section 3.3, hydrogen‐bonded base pairs provide a well‐defined structural environment where proton tunnelling can occur and be modelled computationally and experimentally.

Within this framework, proton tunnelling induces transient tautomeric states that alter base‐pairing fidelity. These events, while short‐lived, can be propagated through replication processes, introducing mutations without external chemical damage. Thus, DNA base pairs function as discrete quantum mutational units, in which quantum fluctuations are translated into heritable genomic variation. This perspective provides a mechanistic basis for spontaneous mutagenesis and its contribution to oncogenesis and genome instability.

#### 3.6.3 A unified molecular framework

Across these systems, a common pattern emerges: quantum effects are not diffusely distributed but are concentrated within specific molecular architectures that act as functional nodes. These nodes are characterised by structurally constrained environments, sensitivity to environmental coupling and measurable quantum parameters such as tunnelling rates or coherence lifetimes.

Importantly, these features define not only how quantum effects operate but also how they can be experimentally accessed and interpreted. As a result, quantum‐associated target molecules provide a tractable bridge between nanoscale quantum behaviour and mesoscale biological outcomes, including metabolic imbalance and mutational accumulation.

By focusing on such target molecules, quantum biology shifts from a descriptive discipline to a mechanism‐oriented and experimentally grounded framework, enabling direct linkage between measurable quantum parameters and disease‐relevant phenotypes without extending beyond biologically and clinically tractable systems.

## 4 EXPERIMENTAL AND COMPUTATIONAL ADVANCES

**FIGURE 2:**
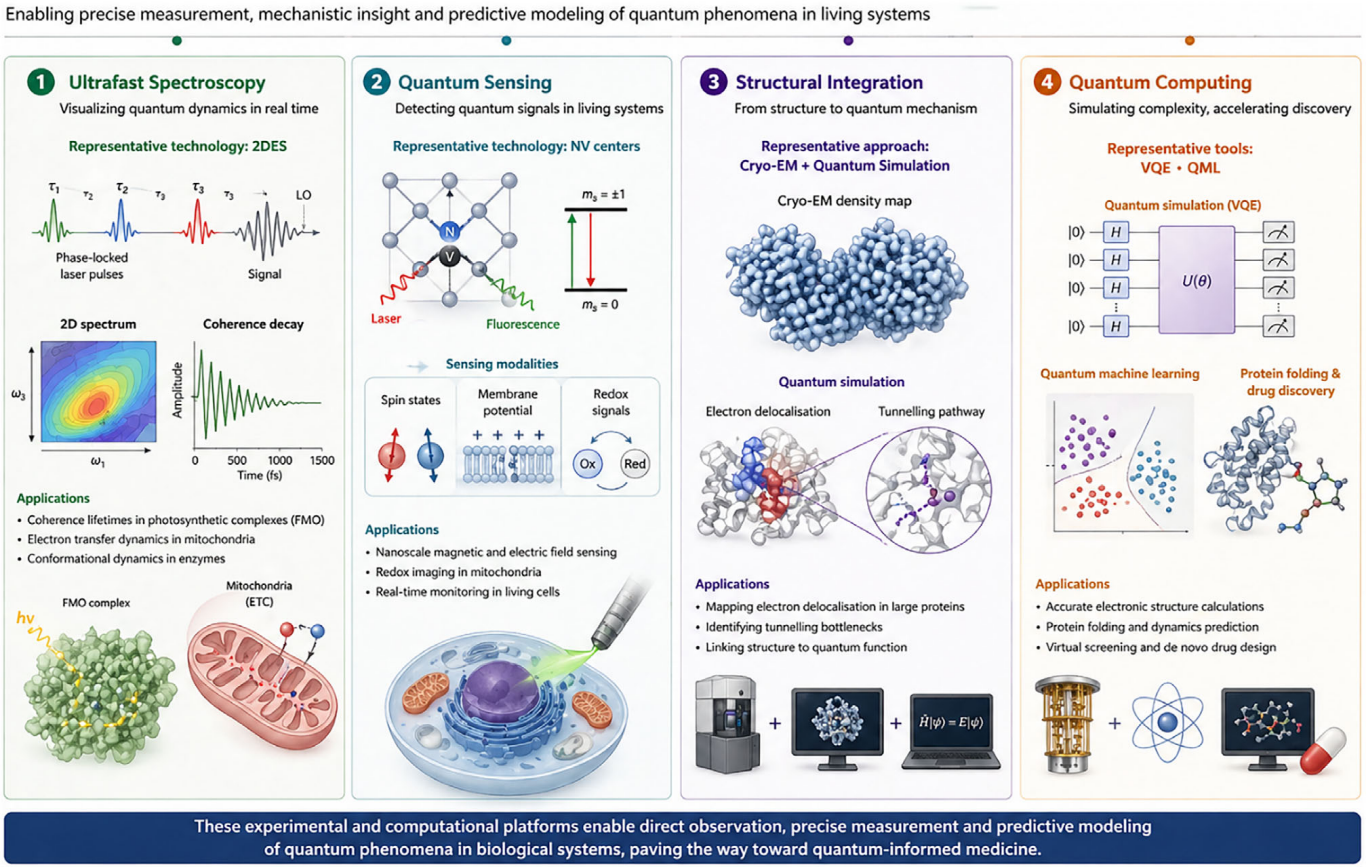
Experimental and computational advances in quantum biology. This figure illustrates four key technologies that are transforming quantum biology into a measurable and applicable science. Ultrafast spectroscopy such as 2DES enables femtosecond‐resolved visualisation of coherence lifetimes and electron dynamics in systems like photosynthetic complexes and mitochondria. Quantum sensing with NV centres in diamond detects spin states, membrane potentials and redox signals in living cells with nanoscale precision. Cryo‐EM provides high‐resolution structural data that, when integrated with quantum simulations, reveals electron delocalisation and tunnelling bottlenecks in large biomolecules. Finally, quantum computing tools like VQE and quantum machine learning are opening new possibilities for simulating complex biomolecular systems, predicting protein folding and accelerating drug discovery. Together, these platforms bring quantum biological phenomena into reach for experimental and translational applications.

Quantum biology has matured from a speculative field into a data‐driven science largely because of transformative advances in experimental measurement and computational modelling. These tools allow direct observation of coherence, tunnelling and spin dynamics in biological systems and provide theoretical frameworks for simulating biomolecular quantum states with unprecedented accuracy. Together, they form the backbone of modern quantum biophysics (Figure 2).

### 4.1 Ultrafast spectroscopy

#### 4.1.1 Two‐dimensional electronic spectroscopy

Ultrafast laser spectroscopy has become indispensable for probing quantum coherence in biomolecules. 2DES provides femtosecond temporal resolution while simultaneously resolving frequency domains, enabling visualisation of coherent oscillations in real time.

Here, ‘coherence oscillations’ refer to time‐dependent oscillatory modulations arising from phase‐coherent superpositions between quantum states, typically manifested as oscillations in the off‐diagonal elements of the density matrix and observed as quantum beat signals in spectroscopic measurements.

By separating ground‐state bleach, stimulated emission and excited‐state absorption signals, 2DES can directly identify quantum beat signatures of excitonic superposition states.^95^, ^96^, ^97^

#### 4.1.2 Applications in photosynthesis

In photosynthetic light‐harvesting complexes, 2DES revealed that excitons remain coherent for hundreds of femtoseconds, even at physiological temperatures. This finding revolutionised our understanding of photosynthetic energy transfer, demonstrating that coherence is not an artifact of cryogenic conditions but a functional strategy for near‐lossless transport.^98^

#### 4.1.3 Applications in mitochondria

More recently, ultrafast spectroscopy has been applied to mitochondrial ETCs, where electron transfer through FMN and Fe–S clusters is thought to involve quantum tunnelling and coherence. These experimental advances enable the direct observation of quantum phenomena by resolving specific measurable signatures. Ultrafast spectroscopic techniques, such as 2DES, capture coherent oscillatory signals in time‐resolved spectra, which are interpreted as signatures of quantum coherence. In parallel, quantum sensing approaches, including NV centres in diamond, detect spin‐dependent signals and local electromagnetic field fluctuations at the nanoscale, providing direct evidence of quantum states and their dynamics in biological systems.

Preliminary studies have shown that mitochondria from tumour cells exhibit shorter coherence lifetimes compared with those from healthy cells, suggesting that quantum decoherence may underlie bioenergetic dysfunction and ROS overproduction in disease contexts.^99^

By enabling direct visualisation of coherence dynamics, 2DES and related ultrafast methods provide experimental validation for quantum‐biological theories and create a bridge between physical predictions and biological function.^100^

### 4.2 Quantum sensing

#### 4.2.1 NV centres in diamond as quantum probes

Quantum sensing technologies based on NV centres in diamond offer nanoscale resolution of magnetic and electric fields under ambient conditions. NV centres exploit the quantum spin states of NV defects, which can be optically initialised and read out with high sensitivity. These defects serve as quantum magnetometers and electrometers, capable of detecting fields generated by single molecules or organelles.^101^, ^102^, ^103^, ^104^

#### 4.2.2 Magnetic resonance and spin detection in cells

In biological systems, NV magnetometry allows visualisation of redox activity, ROS dynamics and mitochondrial membrane potential inside living cells. For example, the spin state of radical pairs in cryptochromes can be probed to study magnetoreception, while ROS generated by dysfunctional mitochondria can be detected in real time at subcellular resolution. NV‐based sensing has thus emerged as a powerful complement to ultrafast spectroscopy, extending quantum measurements into living systems and providing direct connections between quantum dynamics and cellular physiology.^105^

#### 4.2.3 Biomedical potential

Beyond fundamental studies, NV sensing holds promise for non‐invasive diagnostics. By detecting quantum‐level signatures such as altered spin coherence or abnormal redox activity, NV sensors could one day serve as quantum biomarkers for early disease detection, including cancer and neurodegeneration.^106^

### 4.3 Structural integration

While structural biology and quantum computing are broadly applied across many areas of molecular biology, their relevance in this context lies in enabling the direct investigation of quantum phenomena in biological systems. In particular, high‐resolution structural data and quantum‐enabled simulations provide a framework to probe electron transfer, coherence, tunnelling and spin‐dependent processes at the molecular level, thereby linking technological advances to the mechanistic foundations of quantum biology and its biomedical implications.

#### 4.3.1 Cryo‐EM revolution in structural biology

Cryo‐EM now enables determination of large biomolecular assemblies at near‐atomic resolution, overcoming limitations of X‐ray crystallography and NMR. This structural revolution provides the foundation for integrating quantum simulations with experimentally determined conformations.

#### 4.3.2 Coupling cryo‐EM with quantum simulation

For example, cryo‐EM structures of mitochondrial Complex I and cytochrome c oxidase can be combined with VQE or DMRG calculations to model electron‐transfer pathways, charge delocalisation and redox potentials. This hybrid approach enables identification of bottlenecks in electron transport that arise from mutations or conformational changes, directly linking atomic‐level structure to quantum‐level function.^107^, ^108^

#### 4.3.3 Functional insights

Such integrations have already revealed specific Fe–S clusters where electron delocalisation is disrupted in cancer‐associated mutations, explaining impaired bioenergetics. Going forward, structural–quantum integration may guide drug discovery efforts, for instance, by identifying allosteric binding pockets capable of restoring coherence or bypassing defective electron‐transfer routes.^12^, ^109^, ^110^, ^111^

### 4.4 Quantum computing in biology

#### 4.4.1 VQE for biomolecular Hamiltonians

Classical computers struggle to solve the electronic structure of large biomolecules, particularly those with strongly correlated electrons (e.g., Fe–S clusters, flavoproteins). The VQE,^112^ a hybrid quantum–classical scheme, is employed to approximate ground and excited states of molecular Hamiltonians using near‐term quantum processors. Early demonstrations show that VQE can accurately compute redox potentials and coupling constants in Fe–S clusters, directly relevant to mitochondrial electron transfer.^113^

#### 4.4.2 QML in protein folding and ligand binding

Beyond simulation, quantum computing offers new approaches to data‐driven biology. QML methods, such as quantum kernel methods, QSVMs and variational quantum neural networks (VQNNs), are being applied to multi‐omics datasets, protein structure prediction and drug discovery.^114^

In protein folding, algorithms like the quantum approximate optimisation algorithm (QAOA)^115^ can map folding landscapes more efficiently than classical heuristics, identifying metastable intermediates that underlie misfolding diseases such as cancer‐related p53 mutations. In drug discovery, quantum‐enhanced models improve prediction of binding affinities and accelerate de novo molecular design, moving beyond brute‐force screening towards quantum‐guided medicinal chemistry.

#### 4.4.3 Future potential

Although still constrained by the limitations of noisy intermediate‐scale quantum (NISQ) devices, these methods foreshadow a future in which full‐scale quantum simulation of biomolecules becomes routine. Such advances would fundamentally transform our ability to predict, manipulate and engineer biological systems at the quantum level.^116^, ^117^

Experimental and computational advances have transformed quantum biology from theoretical speculation into an empirically grounded field. Ultrafast spectroscopy has confirmed coherence lifetimes in photosynthetic and mitochondrial systems; NV‐based quantum sensing has opened windows into live‐cell spin and redox dynamics; cryo‐EM structures integrated with quantum simulations connect structure to quantum function; and quantum computing provides the tools to solve Hamiltonians and analyse biological data far beyond classical limits. Collectively, these innovations are establishing a new era in which quantum processes in biology are observable, modellable and increasingly exploitable for biomedical applications.^118^, ^119^

## 5 BIOMEDICAL IMPLICATIONS

**FIGURE 3:**
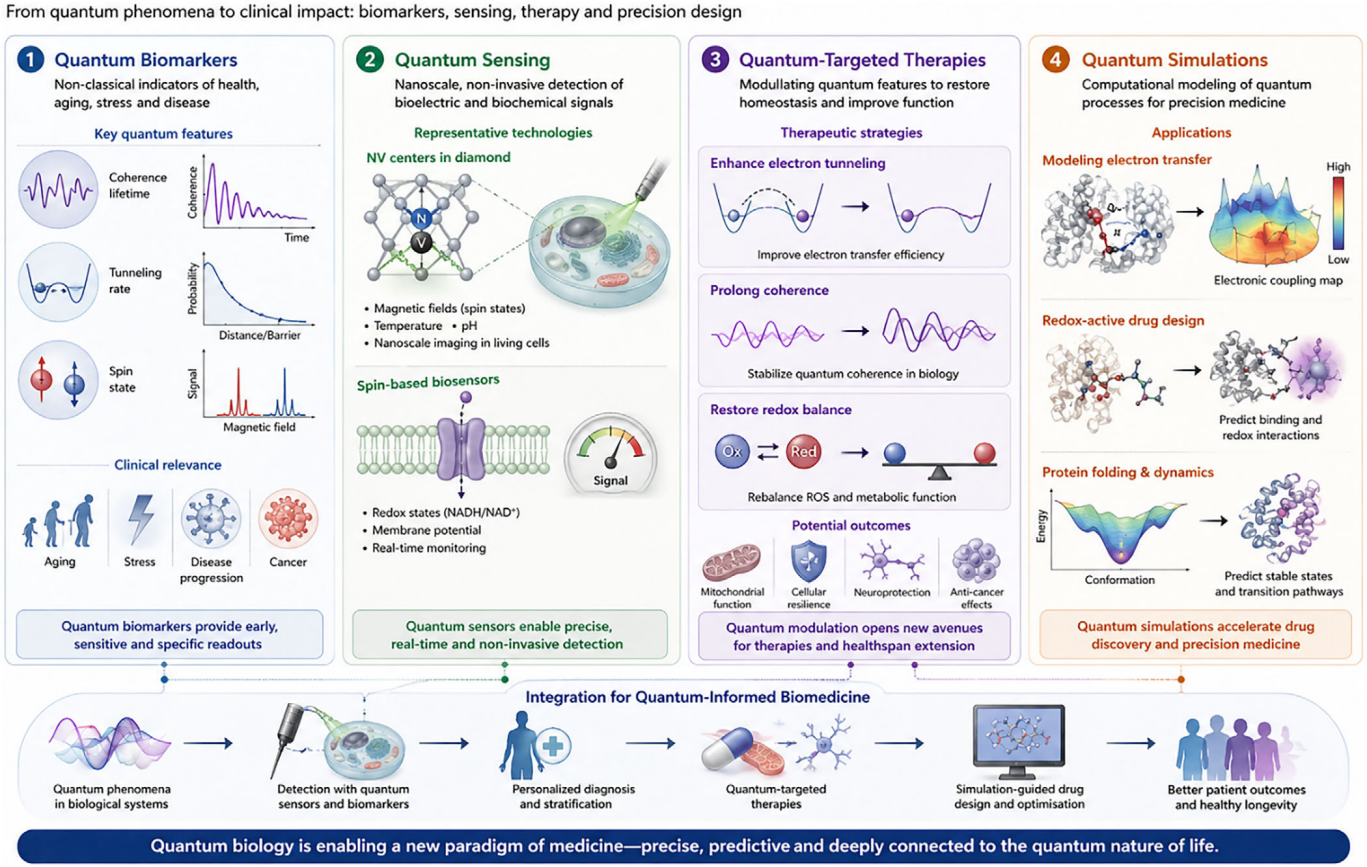
Quantum‐informed diagnostics and therapeutics. This figure illustrates how quantum biology is converging with translational medicine to enable next‐generation diagnostics and therapies. Quantum biomarkers – such as altered coherence lifetimes, tunnelling rates or spin states – offer non‐classical indicators of aging, stress and disease. Quantum sensing technologies, including NV centres and spin‐based biosensors, enable nanoscale, non‐invasive detection of redox states and membrane potentials. On the therapeutic side, modulating quantum features like tunnelling or coherence may restore redox balance or improve metabolic function. Finally, quantum simulations support precision drug design by modelling electron‐transfer pathways and redox‐active interactions. Together, these strategies define a new paradigm of quantum‐informed biomedicine.

The convergence of quantum biology and medicine suggests that quantum phenomena are not only fundamental to life's processes but also central to the onset, progression and treatment of disease. Quantum effects such as coherence, tunnelling and spin dynamics may underpin hallmarks of human health, aging, cognition and pathology. Understanding these processes provides opportunities to identify new biomarkers, reveal disease mechanisms and design quantum‐informed therapies (Figure 3).^120^, ^121^

### 5.1 Aging and metabolism

#### 5.1.1 ROS and mitochondrial decoherence

Mitochondria are key bioenergetic organelles where electron tunnelling and coherence enable efficient ATP production. When coherence is disrupted – through mutations, environmental stress or age‐related damage – electron leakage increases, leading to excess ROS production. While low levels of ROS play signalling roles, chronic accumulation damages DNA, proteins and membranes, driving cellular senescence and organismal aging. From this perspective, mitochondrial decoherence is not merely a metabolic defect but a quantum mechanical failure that accelerates the aging process.^122^, ^123^

#### 5.1.2 Quantum biomarkers of aging

Potential markers of aging may thus include quantum‐level signatures such as shortened coherence lifetimes in ETCs, altered spin states of Fe–S clusters or enhanced proton tunnelling frequencies associated with mutagenesis. Detecting these signatures with ultrafast spectroscopy or NV sensing could provide quantum‐informed biomarkers of biological age, complementing classical measures such as telomere length and epigenetic clocks.^124^

### 5.2 Neurobiology and cognition

#### 5.2.1 Quantum hypotheses in consciousness

The brain represents one of the most intriguing frontiers for quantum biology. The Penrose–Hameroff orchestrated objective reduction^125^ hypothesis proposes that microtubules within neurons sustain quantum coherent states that may contribute to consciousness. While highly debated, this theory highlights the possibility that cognitive processes could involve non‐classical dynamics beyond traditional neurophysiology.^126^

#### 5.2.2 Quantum tunnelling in ion channels and neurotransmission

More concrete evidence suggests that quantum tunnelling plays a role in neuronal signalling. Ion channels, such as potassium and proton channels, may exploit tunnelling to accelerate ion transfer across barriers, ensuring rapid action potential propagation. Neurotransmitter release can be affected by tunnelling effects, since the processes of vesicle fusion and PCET are highly sensitive to dynamics occurring at the quantum scale.^127^, ^128^ These processes could provide the speed and precision required for high‐frequency neural signalling, linking quantum mechanics to cognition and memory.^129^

### 5.3 Disease mechanisms

#### 5.3.1 DNA mutations via tunnelling

Spontaneous mutations arising from proton tunnelling‐induced tautomerisation in DNA bases are implicated in cancer and neurodegeneration. Quantum models predict elevated tunnelling at oncogenic hotspots, consistent with the recurrence of specific point mutations in tumour genomes. In the nervous system, similar tunnelling‐driven mutations may contribute to the accumulation of pathogenic variants linked to disorders such as Alzheimer's or Parkinson's disease.^40^, ^130^

#### 5.3.2 Mitochondrial dysfunction via decoherence

Mitochondria also play a central role in disease through quantum decoherence in their ETCs. Shortened coherence lifetimes correlate with reduced ATP production, elevated ROS and increased apoptosis resistance – all features of cancer cells. Neurodegenerative diseases similarly involve impaired mitochondrial coherence, linking quantum dysfunction to both energy crisis and oxidative stress in neurons. Thus, tunnelling and coherence defects represent quantum origins of pathology across multiple disease categories.^123^

### 5.4 Translational quantum biomedicine

#### 5.4.1 Quantum‐informed diagnostics

**FIGURE 4:**
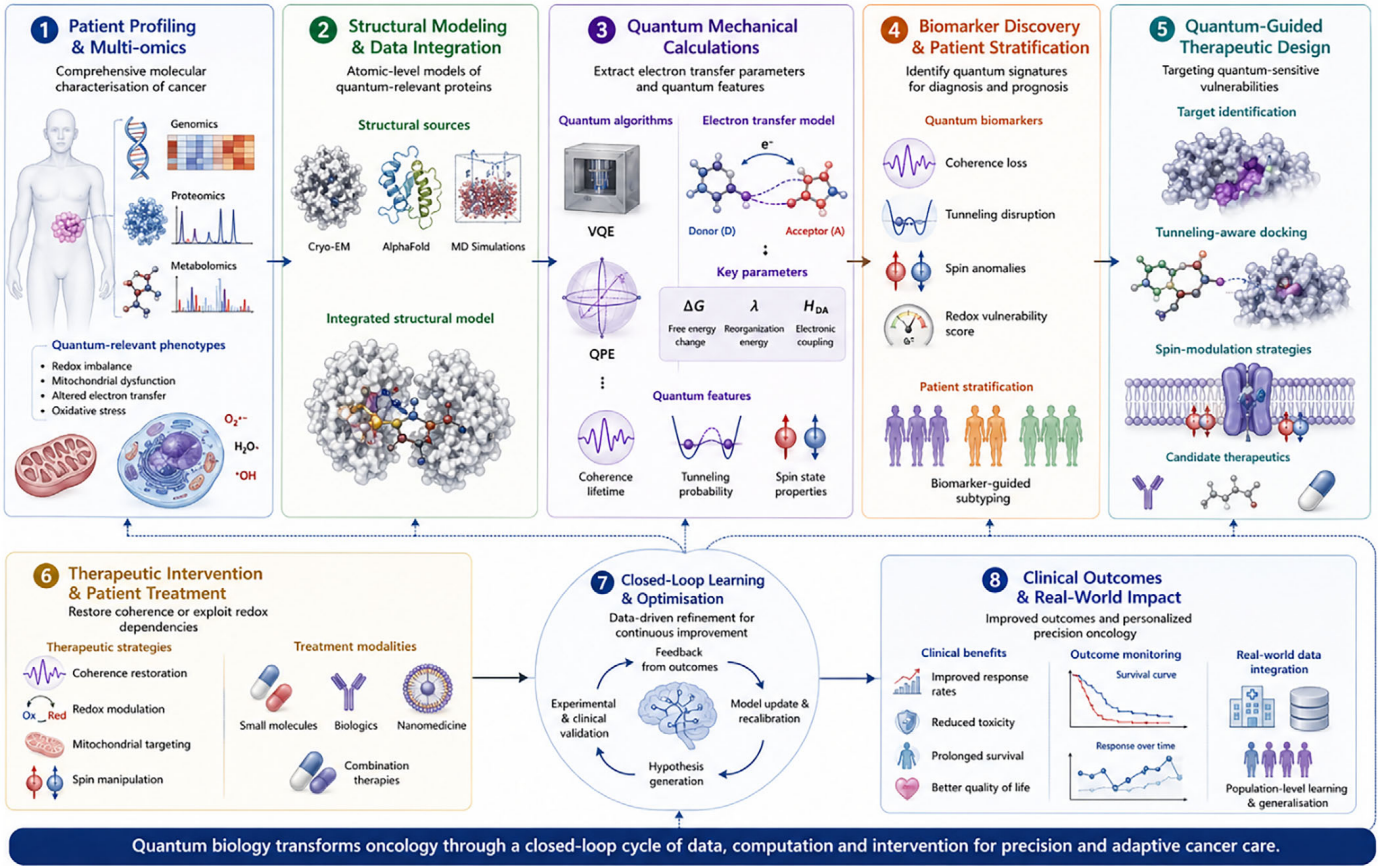
A quantum‐informed oncology workflow. This diagram presents a conceptual framework integrating quantum biology into oncology, from patient profiling to precision therapy. Multi‐omics data – including genomics, proteomics and metabolomics – reveal redox imbalances and mitochondrial dysfunction linked to quantum effects in cancer. Structural models derived from cryo‐EM, AlphaFold or MD simulations support quantum mechanical calculations (e.g., VQE, QPE) to extract key electron transfer parameters such as Δ*G*, *λ* and *H*_DA_. These simulations guide the identification of coherence loss, tunnelling disruption or spin anomalies as diagnostic biomarkers. Drug discovery targets quantum‐sensitive proteins using tunnelling‐aware docking and spin‐modulation strategies. Finally, therapeutic interventions are designed to restore quantum coherence or exploit vulnerabilities in redox signalling. Clinical outcomes feed back into the system, enabling continuous optimisation through a closed‐loop learning framework.

Translational medicine is beginning to incorporate quantum insights into diagnostic pipelines (Figure 4). Ultrafast spectroscopy can reveal altered coherence in mitochondria, NV sensors can map spin dynamics and ROS flux in patient samples, and quantum mutational signatures (e.g., elevated G→A transitions from tunnelling) can refine genomic diagnostics.

Emerging studies suggest that quantum‐informed diagnostic approaches, including measurements of mitochondrial coherence dynamics and redox‐related signatures, may provide additional layers of information for disease characterization. However, these approaches remain at an early stage of development, and further experimental validation is required to establish their clinical utility.

These quantum‐informed biomarkers promise earlier and more precise detection of disease, particularly for cancers and age‐related disorders.^131^

#### 5.4.2 Therapeutics targeting coherence and tunnelling

On the therapeutic front, drugs designed to stabilise electron delocalisation in mitochondrial complexes may restore coherence and reduce ROS generation. On the other hand, by modifying hydration shells or disrupting hydrogen‐bond networks surrounding DNA, certain compounds may inhibit tunnelling‐driven tautomerisation, thereby reducing mutation frequency and potentially slowing the development of cancer. Early studies already test mitochondrial‐targeted antioxidants, spin‐modulating agents and DNA‐stabilising compounds as quantum‐directed therapeutics.^120^, ^132^

#### 5.4.3 The future of quantum‐informed medicine

In the long term, integrating quantum biomarkers with multi‐omics and patient stratification may enable a new paradigm in precision medicine – where therapies are matched not only to genetic and epigenetic profiles but also to the quantum mechanical state of key biomolecular processes.

The biomedical implications of quantum biology are profound. In aging, mitochondrial decoherence accelerates ROS‐driven decline; in neurobiology, tunnelling and coherence may underlie both cognition and disease; in pathology, quantum processes explain mutagenesis and bioenergetic failure; and in translational medicine, quantum‐informed diagnostics and therapeutics are beginning to emerge. Together, these insights suggest that quantum mechanics is not merely compatible with life but integral to its health, dysfunction and treatment.^5^, ^133^, ^134^

## 6 CHALLENGES AND LIMITATIONS

Despite the remarkable progress in quantum biology, significant challenges remain before the field can be fully integrated into mainstream biomedical science. These challenges reflect both fundamental physical constraints and technological bottlenecks, as well as the difficulties of bridging complex theory with experimental reality. Three areas—decoherence in noisy environments, scaling limitations in quantum simulations and the gap between models and validation—represent the most pressing hurdles.^3^

### 6.1 Decoherence in warm, noisy environments

#### 6.1.1 The problem of decoherence

**FIGURE 5:**
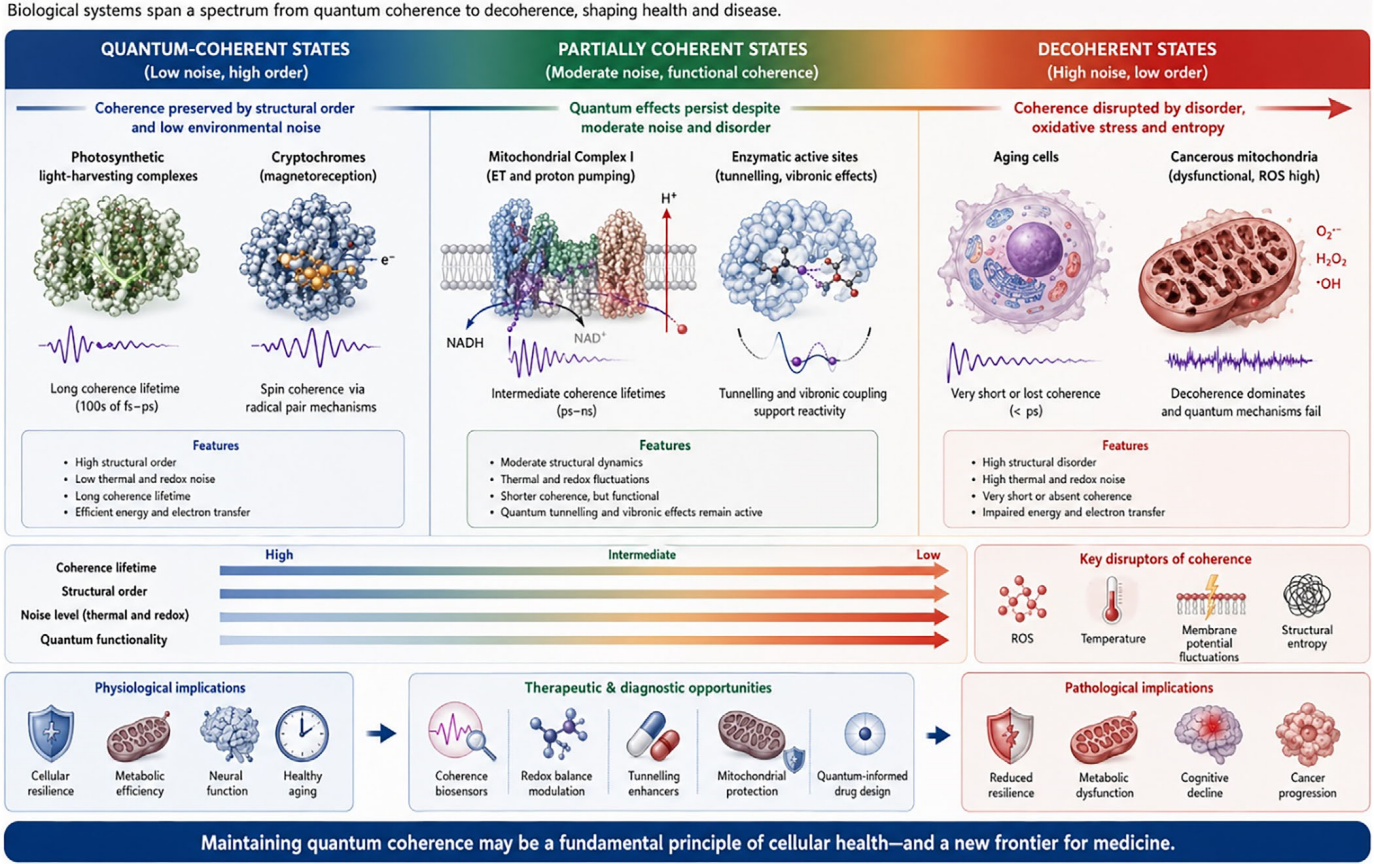
Quantum decoherence landscape in cellular physiology. This diagram illustrates how biological systems occupy a spectrum of quantum coherence. On the left, quantum‐coherent states (e.g., photosynthetic complexes, cryptochromes) preserve coherence through structural order and low noise. The centre depicts partially coherent systems like mitochondrial Complex I and enzymatic active sites, where tunnelling and vibronic effects remain functional despite moderate thermal and redox noise. On the right, decoherent states such as aging cells and cancerous mitochondria suffer from oxidative stress and structural entropy, impairing quantum mechanisms. The landscape suggests that maintaining coherence may be critical for cellular health and introduces novel avenues for quantum‐informed diagnostics and therapies.

Quantum coherence and entanglement are fragile states that rapidly collapse under thermal noise and environmental interactions. Living cells operate at ∼310 K in aqueous, ion‐rich environments that should, in principle, destroy coherence on femtosecond timescales. This raises the central paradox of quantum biology: how can life sustain quantum effects under such inhospitable conditions? (Figure 5).^135^

#### 6.1.2 Partial solutions and open questions

Research indicates that biomolecules could take advantage of ENAQT, a phenomenon in which specific forms of noise help preserve coherence by reducing exciton localisation. Protein scaffolds, vibrational modes and structural symmetry may act as natural error‐correction mechanisms, extending coherence lifetimes beyond naïve predictions. However, these protective strategies remain poorly understood and may vary across systems. Determining the general principles that govern noise‐tolerant quantum states in biology remains a major challenge.

#### 6.1.3 Clinical relevance of decoherence

From a biomedical perspective, decoherence is more than a physical constraint; it may underlie disease. For example, shortened coherence lifetimes in mitochondria have been linked to impaired energy production and elevated ROS in cancer and neurodegeneration. However, proving a direct causal link between decoherence and disease remains challenging, highlighting the necessity for more precise and direct measurement techniques.

### 6.2 Scaling limitations in quantum simulations

#### 6.2.1 The computational bottleneck

Accurately simulating biological molecules at the quantum level requires solving the many‐body Schrödinger equation for systems with thousands of electrons. Classical computers are incapable of handling such complexity beyond small model systems. Even with approximations, traditional quantum chemistry struggles to capture strongly correlated electron dynamics in biomolecules such as Fe–S clusters or photosynthetic reaction centres.

#### 6.2.2 Near‐term quantum hardware

Quantum computing offers a potential breakthrough, but current devices are in the NISQ era. Present quantum processors suffer from short qubit coherence times, limited gate fidelity and insufficient qubit counts to model realistic biomolecules. Algorithms such as the VQE and QAOA have shown promise on small systems, but scaling them to clinically relevant biomolecular networks remains far out of reach.

#### 6.2.3 Path forward

To overcome this limitation, advances are needed in fault‐tolerant quantum hardware, error correction protocols and hybrid quantum–classical workflows that can efficiently allocate computational resources. For now, performing quantum‐level simulations of whole proteins, organelles or cellular signalling pathways is still more of a goal than a reality.

### 6.3 Bridging theoretical models with experimental validation

#### 6.3.1 Theory–experiment disconnect

Many quantum‐biological models predict phenomena—such as long‐lived coherence or tunnelling‐induced mutations – that are difficult to confirm experimentally. Computational predictions often rely on idealised Hamiltonians, whereas biological systems are heterogeneous, dynamic and embedded in complex cellular environments. This creates a persistent gap between theoretical abstraction and biological reality.^136^

#### 6.3.2 Experimental limitations

Although techniques such as ultrafast spectroscopy and NV‐based sensing provide unprecedented resolution, they often probe simplified or isolated systems (purified complexes, in vitro samples) rather than intact living organisms. In addition, because quantum signals are inherently faint, advanced analytical methods are needed to separate them from surrounding noise. Establishing reproducibility and biological relevance across laboratories remains a challenge.

#### 6.3.3 Multidisciplinary integration

Closing this gap requires closer collaboration between quantum physicists, biologists, chemists and clinicians. New computational approaches need to merge quantum mechanics with systems biology and multi‐omics information, whereas experimental studies should create assays that connect quantum phenomena directly to functional outcomes including metabolism, genetic mutations and the course of disease. Without such cross‐disciplinary integration, the clinical translation of quantum biology will remain limited.

Quantum biology faces formidable but surmountable challenges. The fragility of coherence in warm, noisy environments raises fundamental questions about how life protects quantum states. The restrictions of present‐day quantum devices hinder efforts to perform realistic simulations of biomolecules. Finally, the gap between theoretical predictions and experimental validation must be narrowed to ensure that quantum phenomena are more than elegant models—they must be demonstrable, reproducible and clinically meaningful. Addressing these challenges will determine whether quantum biology remains a promising frontier or matures into a transformative pillar of 21st‐century biomedicine.^8^, ^116^, ^137^, ^138^

## 7 FUTURE PERSPECTIVES

Quantum biology is no longer a purely theoretical curiosity – it is emerging as a framework that could reshape our understanding of life and disease.^139^ While these concepts highlight promising directions for the field, many of the proposed mechanisms and applications remain speculative and require further experimental validation. Accordingly, this section is intended to provide a forward‐looking perspective on emerging hypotheses rather than a summary of fully established findings. Looking ahead, the field is poised to evolve in three major directions: the integration of quantum biology into systems‐level approaches, the translation of insights into quantum‐inspired therapies and the formulation of a unified theory of life that explicitly incorporates quantum principles.^8^, ^18^

### 7.1 Integration of quantum biology into systems biology and multi‐omics

#### 7.1.1 From molecules to systems

Current quantum‐biological studies often focus on isolated components—photosynthetic complexes, enzymes or DNA base pairs. Yet, in living organisms, these processes are embedded within highly interconnected molecular networks. The upcoming challenge is to merge quantum‐scale models with systems biology, creating links that span from fundamental subatomic processes to the physiology of entire cells.^122^, ^140^, ^141^

#### 7.1.2 Quantum signatures in omics datasets

Large‐scale genomics, proteomics, metabolomics and epigenomics provide a rich context for correlating quantum signatures with clinical phenotypes. For example, tunnelling‐induced mutational spectra could be linked to tumour sequencing datasets, or coherence lifetimes in mitochondrial proteins could be mapped against transcriptomic and metabolomic changes in aging tissues. Incorporating quantum parameters into multi‐omics integration pipelines could reveal hidden determinants of disease risk and therapeutic response.^16^

Emerging evidence suggests that quantum‐related effects may contribute to complex biological processes and disease‐relevant pathways. However, these associations remain an area of active investigation, and further experimental and mechanistic studies are required to establish their functional significance.

#### 7.1.3 Data‐driven quantum biology

Machine learning and quantum‐enhanced algorithms will play a crucial role in integrating these complex datasets. By leveraging quantum kernel techniques and variational circuits, researchers may identify patterns invisible to classical approaches, thereby building predictive models of health and disease that incorporate quantum‐scale fluctuations. This integration promises to establish quantum biology not as an isolated discipline but as a core dimension of systems biomedicine.^142^

### 7.2 Development of quantum‐inspired therapies

#### 7.2.1 Restoring quantum coherence

As evidence accumulates that decoherence underlies disease, therapies could be designed to stabilise or restore quantum coherence in biomolecular systems. Mitochondrial‐targeted small molecules or peptides that enhance electron delocalization may reduce ROS production, improve energy efficiency and delay aging‐related decline.

#### 7.2.2 Modulating tunnelling pathways

Therapeutics could also be aimed at reducing harmful tunnelling events, such as proton tunnelling that drives DNA mispairing and mutagenesis. Agents that modulate hydration shells, alter proton donor–acceptor geometries, or reinforce canonical hydrogen bonding could lower tunnelling probabilities, thereby reducing mutation rates in cancer or neurodegeneration.

#### 7.2.3 Spin‐based therapies

Another promising direction involves spin chemistry. If radical pair spin dynamics contribute to sensory biology and redox signalling, drugs or magnetic fields designed to influence spin coherence could alter signalling outcomes. Such spin‐modulating interventions may one day represent a new class of quantum‐informed therapeutics.

#### 7.2.4 From concept to clinic

Although speculative, the first clinical trials incorporating quantum biomarkers – such as mitochondrial coherence lifetimes – have already been initiated in oncology. This suggests that quantum‐inspired therapies could transition from concept to reality within the coming decade.

### 7.3 Towards a unified theory of life at the quantum level

#### 7.3.1 Beyond reductionism

Classical biology explains life through genetics, chemistry and thermodynamics, but it leaves unresolved the extraordinary efficiency, precision and adaptability of many biological processes. Quantum biology offers the possibility of a unified theory of life in which coherence, tunnelling and entanglement are recognised as fundamental principles alongside Darwinian evolution and molecular genetics.

#### 7.3.2 Philosophical and scientific implications

Such a theory would not only deepen our understanding of how life works but also redefine our conception of what life is. If quantum processes are essential to biological function, then the origin of life itself may be inseparable from the conditions that support quantum coherence. Moreover, the study of cognition, consciousness and complex behaviour may ultimately require a quantum‐informed neuroscience.

#### 7.3.3 Long‐term vision

In the next 10–20 years, as fault‐tolerant quantum computing matures, researchers may be able to simulate entire metabolic pathways and signalling networks at the electronic level. Entanglement‐based models could be applied to study chromatin dynamics, neural synchrony or even consciousness. The ultimate goal would be a quantum theory of biology that unifies molecular physics with systems‐level physiology, providing a conceptual foundation as transformative as the central dogma or the genetic code.

The advancement of quantum biology will depend on expanding its scale, applying its insights across disciplines and integrating its diverse approaches. By embedding quantum effects into systems biology and multi‐omics frameworks, developing therapies that exploit or correct quantum mechanisms and formulating a unified theory of life grounded in quantum principles, the field has the potential to fundamentally reshape medicine and biology. What began as an exploration of coherence in photosynthetic complexes is now moving towards a vision in which the quantum nature of life is a cornerstone of 21st‐century science and medicine.

## 8 CONCLUSION

1. Quantum biology reveals that life operates through a dual classical–quantum framework, where coherence, tunnelling, spin dynamics and entanglement actively shape core biological processes‐from photosynthetic energy transfer to enzymatic catalysis, DNA stability, sensory perception, mitochondrial bioenergetics and possibly cognition.
2. Quantum mechanisms are not rare anomalies but evolutionary design principles that enable biological systems to achieve efficiencies, precision and adaptability that are unattainable by classical physics alone.
3. Coherence and tunnelling provide mechanistic clarity to long‐standing biological questions, explaining near‐lossless exciton transport, rapid enzyme catalysis and the spontaneous tautomerisation‐driven mutations that influence evolution, aging and disease.
4. Spin dynamics and quantum entanglement offer a unifying explanation for extraordinarily sensitive biological sensing, including magnetoreception and potentially other yet‐undiscovered signalling modalities.
5. Quantum biology provides new biomedical opportunities, including biomarkers such as mitochondrial coherence lifetimes, tunnelling‐associated mutational signatures and spin‐state indicators of redox imbalance and disease progression.
6. Therapeutic innovation may emerge by directly targeting quantum‐level dysfunction, such as restoring electron delocalisation in mitochondria, suppressing pathological proton tunnelling in DNA or modulating spin‐dependent radical pair reactions.
7. Integrating quantum parameters into systems biology and multi‐omics frameworks could uncover hidden determinants of health, aging and disease that remain invisible to classical molecular analyses.
8. Rapid advances in ultrafast spectroscopy, quantum sensing, cryo‐EM/quantum simulation and quantum computing will accelerate the transition of quantum biology from a foundational science to an applied medical discipline.
9. Quantum biology marks a paradigm shift comparable to the discovery of the genetic code, redefining the fundamental nature of survival, adaptation and disease through the quantum fabric of living matter.
10. By embracing the quantum dimension of life, biology and medicine move towards a future in which diagnostics, therapeutics and systems‐level understanding are grounded not only in chemistry and genetics but also in the quantum architecture that underlies all biological function.

## AUTHOR CONTRIBUTIONS

J. Y. S. wrote the original draft. J. Y. S. and J. H. C. designed and wrote the paper. Both authors contributed to the interpretation of the results. Both authors have read and agreed to the published version of the manuscript.

## CONFLICT OF INTEREST STATEMENT

The authors declare no conflicts of interest.

## ETHICS STATEMENT

None.

## References

[1] Babcock N . Open quantum systems theory of ultraweak ultraviolet photon emissions: revisiting Gurwitsch's onion experiment as a prototype for quantum biology. Comput Struct Biotechnol J. 2024;26:78‐91. 39717158 10.1016/j.csbj.2024.11.030 PMC11664013

[2] Slocombe L , Sacchi M , Al‐Khalili J . An open quantum systems approach to proton tunnelling in DNA. Commun Phys. 2022;5(1):109.

[3] Marais A , Adams B , Ringsmuth AK , et al. The future of quantum biology. J R Soc Interface. 2018;15(148):20180640. 30429265 10.1098/rsif.2018.0640 PMC6283985

[4] Lambert N , Chen Y , Cheng Y , Li C , Chen G , Nori F . Quantum biology. Nat Phys. 2012;9(1):10‐18.

[5] Kim Y , Bertagna F , D'Souza EM , et al. Quantum biology: an update and perspective. Quantum Rep. 2021;3(1):80‐126.

[6] Mazzoccoli G . Chronobiology meets quantum biology: a new paradigm overlooking the horizon? Front Physiol. 2022;13:892582. 35874510 10.3389/fphys.2022.892582 PMC9296773

[7] Arndt M , Juffmann T , Vedral V , Quantum physics meets biology. HFSP J. 2010;3(6):386‐400. 10.2976/1.3244985 PMC2839811 20234806

[8] Alvarez PH , Gerhards L , Solov'yov IA , de Oliveira MC . Quantum phenomena in biological systems. Front Quantum Sci Technol. 2024;3:1466906.

[9] Runeson JE , Lawrence JE , Mannouch JR , Richardson JO . Explaining the efficiency of photosynthesis: quantum uncertainty or classical vibrations? J Phys Chem Lett. 2022;13(15):3392‐3399. 35404611 10.1021/acs.jpclett.2c00538 PMC9036581

[10] Higgins JS , Lloyd LT , Sohail SH , et al. Photosynthesis tunes quantum‐mechanical mixing of electronic and vibrational states to steer exciton energy transfer. Proc Natl Acad Sci USA. 2021;118(11):e2018240118. 33688046 10.1073/pnas.2018240118 PMC7980405

[11] Engel GS , Calhoun TR , Read EL , et al. Evidence for wavelike energy transfer through quantum coherence in photosynthetic systems. Nature. 2007;446(7137):782‐786. 17429397 10.1038/nature05678

[12] Hayashi T , Stuchebrukhov A . Electron tunneling in respiratory complex I. Proc Natl Acad Sci USA. 2010;107(45):19157‐19162. 20974925 10.1073/pnas.1009181107 PMC2984193

[13] Page CC , Moser CC , Chen X , Dutton PL . Natural engineering principles of electron tunnelling in biological oxidation–reduction. Nature. 1999;402(6757):47‐52. 10573417 10.1038/46972

[14] Gray H , Winkler J . Long‐range electron transfer. Proc Natl Acad Sci USA. 2005;102(10):3534‐3539. 15738403 10.1073/pnas.0408029102 PMC553296

[15] Martin D , Matyushov D . Electron‐transfer chain in respiratory complex I. Sci Rep. 2017;7(1):5490. 28710385 10.1038/s41598-017-05779-y PMC5511282

[16] Goryanin I , Ivanov Y , Damms B , Vesnin S , Shevelev O , Goryanin I . Exploring the interface between quantum biology, microwave technology, and neuroscience. Drug Discov Today. 2025;30(7):104408. 40513771 10.1016/j.drudis.2025.104408

[17] Maffei M . Plant quantum biology: the quantum dimension of plant responses to stress. Plant Stress. 2025;17:100930.

[18] Cao J , Cogdell RJ , Coker DF , et al. Quantum biology revisited. Sci Adv. 2020;6(14):eaaz4888. 32284982 10.1126/sciadv.aaz4888 PMC7124948

[19] Duan H , Stevens AL , Nalbach P , Thorwart M , Prokhorenko VI , Miller RJD . Two‐dimensional electronic spectroscopy of light‐harvesting complex II at ambient temperature: a joint experimental and theoretical study. J Phys Chem B. 2015;119(36):12017‐12027. 26301382 10.1021/acs.jpcb.5b05592

[20] Schroedinger E . What is life? The physical aspect of the living cell. Cambridge University Press, 1944.

[21] Phillips R . Schrodinger's what is life? at 75. Cell Syst. 2021;12(6):465‐476. 34139159 10.1016/j.cels.2021.05.013 PMC12107503

[22] Duan H , Prokhorenko VI , Cogdell RJ , et al. Nature does not rely on long‐lived electronic quantum coherence for photosynthetic energy transfer. Proc Natl Acad Sci USA. 2017;114(32):8493‐8498. 28743751 10.1073/pnas.1702261114 PMC5559008

[23] Panitchayangkoon G , Hayes D , Fransted KA , et al. Long‐lived quantum coherence in photosynthetic complexes at physiological temperature. Proc Natl Acad Sci USA. 2010;107(29):12766‐12770. 20615985 10.1073/pnas.1005484107 PMC2919932

[24] Caycedo‐Soler F , Mattioni A , Lim J , Renger T , Huelga SF , Plenio MB . Exact simulation of pigment‐protein complexes unveils vibronic renormalization of electronic parameters in ultrafast spectroscopy. Nat Commun. 2022;13(1):2912. 35614049 10.1038/s41467-022-30565-4 PMC9133012

[25] Ishizaki A , Fleming G , Theoretical examination of quantum coherence in a photosynthetic system at physiological temperature. Proc Natl Acad Sci USA. 2009;106(41):17255‐17260. 19815512 10.1073/pnas.0908989106 PMC2762676

[26] Löwdin P , Proton tunneling in DNA and its biological implications. Rev Mod Phys. 1963;35(3):724‐732.

[27] Shekaari A , Jafari M , Modeling the action of environment on proton tunneling in the adenine–thymine base pair. Prog Biophys Mol Biol. 2020;150:98‐103. 31299278 10.1016/j.pbiomolbio.2019.07.002

[28] Parey K , Lasham J , Mills DJ , et al. High‐resolution structure and dynamics of mitochondrial complex I—Insights into the proton pumping mechanism. Sci Adv. 2021;7(46):eabj3221. 34767441 10.1126/sciadv.abj3221 PMC8589321

[29] Lim H , et al., Fragment molecular orbital‐based variational quantum eigensolver for quantum chemistry in the age of quantum computing. Sci Rep. 2024;14(1):2422. 38287087 10.1038/s41598-024-52926-3 PMC10825197

[30] Liu L , Zou Q , Leung J , et al. Ultrafast labeling and high‐fidelity imaging of mitochondria in cancer cells using an aggregation‐enhanced emission fluorescent probe. Chem Commun. 2019;55(97):14681‐14684. 10.1039/c9cc07775h 31746860

[31] Nie L , Nusantara AC , Damle VG , et al., Quantum monitoring of cellular metabolic activities in single mitochondria. Sci Adv. 2021;7(21):eabf0573. 34138746 10.1126/sciadv.abf0573 PMC8133708

[32] Wang N , Cai J . Hybrid quantum sensing in diamond. Front Phys. 2024;12:1387193.

[33] Doga H , Raubenolt B , Cumbo F , Joshi J , DiFilippo FP , Qin J , Blankenberg D , Shehab O . A perspective on protein structure prediction using quantum computers. J Chem Theory Comput. 2024;20(9):3359‐3378. 38703105 10.1021/acs.jctc.4c00067 PMC11099973

[34] Lorenzoni N , Lacroix T , Lim J , Tamascelli D , Huelga SF , Plenio MB . Full microscopic simulations uncover persistent quantum effects in primary photosynthesis. Sci Adv. 2025;11(40):eady6751. 41032605 10.1126/sciadv.ady6751 PMC13155558

[35] Dwiputra D , Zen F . Environment‐assisted quantum transport and mobility edges. Phys Rev A. 2021;104(2):022205.

[36] Zerah‐Harush E , Dubi Y . Effects of disorder and interactions in environment assisted quantum transport. Phys Rev Res. 2020;2(2):023294.

[37] Klinman J , Kohen A . Hydrogen tunneling links protein dynamics to enzyme catalysis. Annu Rev Biochem. 2013;82(1):471‐496. 23746260 10.1146/annurev-biochem-051710-133623 PMC4066974

[38] Trixler F . Quantum tunnelling to the origin and evolution of life. Curr Org Chem. 2013;17(16):1758‐1770. 24039543 10.2174/13852728113179990083 PMC3768233

[39] Moser CC , Keske JM , Warncke K , Farid RS , Dutton PL . Nature of biological electron transfer. Nature. 1992;355(6363):796‐802. 1311417 10.1038/355796a0

[40] Srivastava R . The role of proton transfer on mutations. Front Chem. 2019;7:536. 31497591 10.3389/fchem.2019.00536 PMC6712085

[41] Pusuluk O , Farrow T , Deliduman C , Burnett K , Vedral V . Proton tunnelling in hydrogen bonds and its implications in an induced‐fit model of enzyme catalysis. Proc R Soc A. 2018. 474(2218):20180037.

[42] Sutcliffe MJ , Masgrau L , Roujeinikova A , et al. Hydrogen tunnelling in enzyme‐catalysed H‐transfer reactions: flavoprotein and quinoprotein systems. Philos Trans R Soc L B Biol Sci. 2006;361(1472):1375‐1386. 10.1098/rstb.2006.1878 PMC1647315 16873125

[43] Bahnson BJ , Colby TD , Chin JK , Goldstein BM , Klinman JP . A link between protein structure and enzyme catalyzed hydrogen tunneling. Proc Natl Acad Sci USA. 1997;94(24):12797‐12802. 9371755 10.1073/pnas.94.24.12797 PMC24218

[44] Slocombe L , Winokan M , Al‐Khalili J , Sacchi M . Proton transfer during DNA strand separation as a source of mutagenic guanine‐cytosine tautomers. Commun Chem. 2022;5(1):144. 36697962 10.1038/s42004-022-00760-x PMC9814255

[45] Pushkaran A , Arabi A . A review on point mutations via proton transfer in DNA base pairs in the absence and presence of electric fields. Int J Biol Macromol. 2024;277:134051. 39069038 10.1016/j.ijbiomac.2024.134051

[46] Klinman J . The role of tunneling in enzyme catalysis of C–H activation. Biochim Biophys Acta (BBA)—Bioenerg. 2006;1757(8):981‐987. 10.1016/j.bbabio.2005.12.004 16546116

[47] Guo L , Xu B‐M , Zou J , Shao B . Quantifying magnetic sensitivity of radical pair based compass by quantum fisher information. Sci Rep. 2017;7(1):5826. 28725054 10.1038/s41598-017-06187-y PMC5517522

[48] Kattnig D , Hore P , The sensitivity of a radical pair compass magnetoreceptor can be significantly amplified by radical scavengers. Sci Rep. 2017;7(1):11640. 28912470 10.1038/s41598-017-09914-7 PMC5599710

[49] Thoradit T , Thongyoo K , Kamoltheptawin K , et al. Cryptochrome and quantum biology: unraveling the mysteries of plant magnetoreception. Front Plant Sci. 2023;14:1266357. 37860259 10.3389/fpls.2023.1266357 PMC10583551

[50] Zhang Y , Berman G , Kais S , The radical pair mechanism and the avian chemical compass: quantum coherence and entanglement. Int J Quantum Chem. 2015;115(19):1327‐1341.

[51] Matysik J , Gerhards L , Theiss T , et al. Spin dynamics of flavoproteins. Int J Mol Sci. 2023;24(9):8067. 37175925 10.3390/ijms24098218 PMC10179055

[52] Usselman RJ , Chavarriaga C , Castello PR , et al. The quantum biology of reactive oxygen species partitioning impacts cellular bioenergetics. Sci Rep. 2016;6(1):38543. 27995996 10.1038/srep38543 PMC5172244

[53] Austvold CK , Keable SM , Procopio M , Usselman RJ . Quantitative measurements of reactive oxygen species partitioning in electron transfer flavoenzyme magnetic field sensing. Front Physiol. 2024;15:1394093. 10.3389/fphys.2024.1348395 PMC10869518 38370016

[54] Hore P . Spin chemistry in living systems. Natl Sci Rev. 2024;11(9):nwae126. 39144744 10.1093/nsr/nwae126 PMC11321246

[55] Zadeh‐Haghighi H , Simon C . Magnetic field effects in biology from the perspective of the radical pair mechanism. J R Soc Interface. 2022;19(193):20220325. 35919980 10.1098/rsif.2022.0325 PMC9346374

[56] Sarovar M , Ishizaki A , Fleming GR , Whaley KB . Quantum entanglement in photosynthetic light‐harvesting complexes. Nat Phys. 2010;6(6):462‐467.

[57] Olson J . The FMO Protein. Photosynth Res. 2004;80(1‐3):181‐187. 16328820 10.1023/B:PRES.0000030428.36950.43

[58] Ishizaki A , Fleming G . Theoretical examination of quantum coherence in a photosynthetic system at physiological temperature. Proc Natl Acad Sci USA. 2009;106(41):17255‐17260. 19815512 10.1073/pnas.0908989106 PMC2762676

[59] Zerah Harush E , Dubi Y . Do photosynthetic complexes use quantum coherence to increase their efficiency? Probably not. Sci Adv. 2021;7(8):eabc4631. 33597236 10.1126/sciadv.abc4631 PMC7888942

[60] Tang H , Shang X , Shi Z , et al. Simulating photosynthetic energy transport on a photonic network. npj Quantum Inf. 2024;10(1):34.

[61] Panitchayangkoon G , Hayes D , Fransted KA , et al. Long‐lived quantum coherence in photosynthetic complexes at physiological temperature. Proc Natl Acad Sci USA. 2010;107(29):12766‐12770. 20615985 10.1073/pnas.1005484107 PMC2919932

[62] Mohseni M , Rebentrost P , Lloyd S , Aspuru‐Guzik A . Environment‐assisted quantum walks in photosynthetic energy transfer. J Chem Phys. 2008;129(17):174106. 19045332 10.1063/1.3002335

[63] Yue S , Wang Z , Leng X , Zhu R , Chen H , Weng Y . Coupling of multi‐vibrational modes in bacteriochlorophyll a in solution observed with 2D electronic spectroscopy. Chem Phys Lett. 2017;683:591‐597.

[64] Klinman J , Kohen A . Hydrogen tunneling links protein dynamics to enzyme catalysis. Annu Rev Biochem. 2013;82:471‐496. 23746260 10.1146/annurev-biochem-051710-133623 PMC4066974

[65] Klinman J . Linking protein structure and dynamics to catalysis: the role of hydrogen tunnelling. Philos Trans R Soc B: Biol Sci. 2006;361(1472):1323‐1331. 10.1098/rstb.2006.1870 PMC1647309 16873120

[66] Cha Y , Murray C , Klinman J . Hydrogen tunneling in enzyme reactions. Science. 1989;243(4896):1325‐1330. 2646716 10.1126/science.2646716

[67] Jevtic S , Anders J . A qualitative quantum rate model for hydrogen transfer in soybean lipoxygenase. J Chem Phys. 2017;147(11):115101. 28938801 10.1063/1.4998941

[68] Álvarez‐Barcia S , Kästner J . Atom tunneling in the hydroxylation process of taurine/α‐ketoglutarate dioxygenase identified by quantum mechanics/molecular mechanics simulations. J Phys Chem B. 2017;121(21):5347‐5354. 28490178 10.1021/acs.jpcb.7b03477

[69] Sen A , Kohen A . Enzymatic tunneling and kinetic isotope effects: chemistry at the crossroads. J Phys Org Chem. 2010;23(7):613‐619.

[70] Klinman J . An integrated model for enzyme catalysis emerges from studies of hydrogen tunneling. Chem Phys Lett. 2009;471(4‐6):179‐193. 20354595 10.1016/j.cplett.2009.01.038 PMC2846846

[71] Austin A , Sager J , Phan L , Lu Y . Structural effects on the hydride‐tunneling kinetic isotope effects of NADH/NAD+ model reactions: relating to the donor–acceptor distances. J Org Chem. 2025;90(8):3110‐3115. 39946088 10.1021/acs.joc.4c03080 PMC11877500

[72] Yahashiri A , Rubach J , Plapp B . Effects of cavities at the nicotinamide binding site of liver alcohol dehydrogenase on structure, dynamics and catalysis. Biochemistry.2014;53(5):881‐894. 24437493 10.1021/bi401583f PMC3969020

[73] Odai K , Umesaki K . Kinetic study of transition mutations from G–C to A–T base pairs in Watson–Crick DNA base pairs: double proton transfers. J Phys Chem A. 2021;125(37):8196‐8204. 34516113 10.1021/acs.jpca.1c05604

[74] Umesaki K , Odai K . A kinetic approach to double proton transfer in Watson–Crick DNA base pairs. J Phys Chem B. 2020. 10.1021/acs.jpcb.9b11874 32045241

[75] Luo J . Sub‐picosecond proton tunnelling in deformed DNA hydrogen bonds under an asymmetric double‐oscillator model. Eur Phys J E. 2018;41(7):88. 29974268 10.1140/epje/i2018-11690-y

[76] Fedeles B , Li D , Singh V . Structural insights into tautomeric dynamics in nucleic acids and in antiviral nucleoside analogs. Front Mol Biosci. 2021;8:823253. 35145998 10.3389/fmolb.2021.823253 PMC8822119

[77] Matarèse BFE , Rusin A , Seymour C , Mothersill C . Quantum biology and the potential role of entanglement and tunneling in non‐targeted effects of ionizing radiation: a review and proposed model. Int J Mol Sci. 2023;24(22):16157. 38003655 10.3390/ijms242216464 PMC10671017

[78] Turin L . A spectroscopic mechanism for primary olfactory reception. Chem Senses. 1996;21(6):773‐791. 8985605 10.1093/chemse/21.6.773

[79] Hoehn RD , Nichols DE , Neven H , Kais S . Status of the vibrational theory of olfaction. Front Phys. 2018;6:25.

[80] Block E , Jang S , Matsunami H , et al. Implausibility of the vibrational theory of olfaction. Proc Natl Acad Sci USA. 2015;112(21):E2766–E2774. 25901328 10.1073/pnas.1503054112 PMC4450420

[81] Tirandaz A , Taher Ghahramani F , and Salari V . Validity examination of the dissipative quantum model of olfaction. Sci Rep. 2017;7(1):41614. 28667321 10.1038/s41598-017-04846-8 PMC5493690

[82] Hiscock HG , Worster S , Kattnig DR , et al. The quantum needle of the avian magnetic compass. Proc Natl Acad Sci USA. 2016;113(17):4634‐4639. 27044102 10.1073/pnas.1600341113 PMC4855607

[83] Ritz T , Adem S , Schulten K . A model for photoreceptor‐based magnetoreception in birds. Biophys J. 2000. 78(2):707‐718. 10653784 10.1016/S0006-3495(00)76629-X PMC1300674

[84] Bandyopadhyay J , Paterek T , Kaszlikowski D . Quantum coherence and sensitivity of avian magnetoreception. Phys Rev Lett. 2012;109(11). 10.1103/PhysRevLett.109.110502 23005606

[85] Hogben H , Biskup T , Hore P . Entanglement and sources of magnetic anisotropy in radical pair‐based avian magnetoreceptors. Phys Rev Lett. 2012;109(22):110502. 23368109 10.1103/PhysRevLett.109.220501

[86] Stoneham A , Gauger E , Porfyrakis K , Benjamin S , Lovett B . A new type of radical‐pair‐based model for magnetoreception. Biophys J. 2012;102(5):961‐968. 22404918 10.1016/j.bpj.2012.01.007 PMC3296028

[87] Costa G , Liang R . Decrypting the nonadiabatic photoinduced electron transfer mechanism in light‐sensing cryptochrome. ACS Cent Sci. 2025;11(7):1071‐1082. 40726782 10.1021/acscentsci.5c00376 PMC12291142

[88] Pokorný J , Pokorný J , Foletti A , Kobilková J , Vrba J , Vrba J . Mitochondrial dysfunction and disturbed coherence: gate to cancer. Pharm (Basel). 2015;8(4):675‐695. 10.3390/ph8040675 PMC4695805 26437417

[89] Zong Y , Li H , Liao P , et al. Mitochondrial dysfunction: mechanisms and advances in therapy. Signal Transduct Target Ther. 2024;9(1):300. 38744846 10.1038/s41392-024-01839-8 PMC11094169

[90] Egg M , Kietzmann T . Little strokes fell big oaks: the use of weak magnetic fields and reactive oxygen species to fight cancer. Redox Biol. 2025;79:103512. 10.1016/j.redox.2024.103483 PMC11733197 39729909

[91] Dai D , Chiao YA , Marcinek DJ , Szeto HH , Rabinovitch PS . Mitochondrial oxidative stress in aging and healthspan. Longev Heal. 2014;3(1):6. 10.1186/2046-2395-3-6 PMC4013820 24860647

[92] Bhatti J , Bhatti G , Reddy P . Mitochondrial dysfunction and oxidative stress in metabolic disorders — A step towards mitochondria based therapeutic strategies. Biochim Biophys Acta (BBA)—Mol Basis Dis. 2017;1863(5):1066‐1077. 10.1016/j.bbadis.2016.11.010 PMC5423868 27836629

[93] Scialò F , Fernández‐Ayala D , Sanz A . Role of mitochondrial reverse electron transport in ROS signaling: potential roles in health and disease. Front Physiol. 2017;8:428. 28701960 10.3389/fphys.2017.00428 PMC5486155

[94] Seneff S , Zaminpira S , Niknamian S . Quantum entanglement in theoretical physics as a new insight into cancer biology. Afr J Biol Sci. 2019;01(02):1‐12.

[95] Biswas S , Kim J , Zhang X , Scholes GD . Coherent two‐dimensional and broadband electronic spectroscopies. Chem Rev. 2022;122(3):4257‐4321. 35037757 10.1021/acs.chemrev.1c00623

[96] Kim J , Jeon J , Yoon TH , Cho M . Two‐dimensional electronic spectroscopy of bacteriochlorophyll a with synchronized dual mode‐locked lasers. Nat Commun. 2020;11(1):271. 33247112 10.1038/s41467-020-19912-5 PMC7699642

[97] Guimarães J , Lim J , Vasilevskiy MI , Huelga SF , Plenio MB . Accelerating two‐dimensional electronic spectroscopy simulations with a probe qubit protocol. Phys Rev Res. 2025;7(2):023130.

[98] Schlau‐Cohen G , Ishizaki A , Fleming G . Two‐dimensional electronic spectroscopy and photosynthesis: fundamentals and applications to photosynthetic light‐harvesting. Chem Phys. 2011;386(1‐3):1‐22.

[99] Read AD , Bentley RE , Archer SL , Dunham‐Snary KJ . Mitochondrial iron‐sulfur clusters: structure, function, and an emerging role in vascular biology. Redox Biol. 2021;47:102164. 34656823 10.1016/j.redox.2021.102164 PMC8577454

[100] Lambrev P , Akhtar P , Tan H . Insights into the mechanisms and dynamics of energy transfer in plant light‐harvesting complexes from two‐dimensional electronic spectroscopy. Biochim Biophys Acta (BBA)—Bioenerg. 2020;1861(4):148064. 10.1016/j.bbabio.2019.07.005 31326408

[101] Fan S , Lopez Llorens L , Perona Martinez FP , Schirhagl R . Quantum sensing of free radical generation in mitochondria of human keratinocytes during UVB exposure. ACS Sens. 2024;9(5):2440‐2446. 38743437 10.1021/acssensors.4c00118 PMC11129351

[102] Mzyk A , Sigaeva A , Schirhagl R . Relaxometry with nitrogen vacancy (NV) centers in diamond. Acc Chem Res.2022;55(24):3572‐3580. 36475573 10.1021/acs.accounts.2c00520 PMC9774663

[103] Zhang J , Ma L , Hou Y , et al. Nanodiamond‐Based Sensing: a revolution for biosensors in capturing elusive bio‐signals in living cells. Adv Drug Deliv Rev. 2025;221:115252. 10.1016/j.addr.2025.115590 40246241

[104] Perona Martínez F , Nusantara AC , Chipaux M , Padamati SK , Schirhagl R . Nanodiamond relaxometry‐based detection of free‐radical species when produced in chemical reactions in biologically relevant conditions. ACS Sens. 2020;5(12):3862‐3869. 33269596 10.1021/acssensors.0c01037 PMC8651177

[105] Nie L , Nusantara AC , Damle VG , et al. Quantum monitoring of cellular metabolic activities in single mitochondria. Sci Adv. 2021;7(21):eabf0573. 34138746 10.1126/sciadv.abf0573 PMC8133708

[106] Segawa T , Igarashi R . Nanoscale quantum sensing with nitrogen‐vacancy centers in nanodiamonds—A magnetic resonance perspective. Prog Nucl Magn Reson Spectrosc. 2023;134–135:20‐38. 10.1016/j.pnmrs.2022.12.001 37321756

[107] Kühlbrandt W , Carreira L , Yildiz Ö . Cryo‐EM of mitochondrial complex I and ATP synthase. Annu Rev Biophys. 2025;54(1):209‐226. 40327437 10.1146/annurev-biophys-060724-110838

[108] Sanchez‐Martinez A , Agip AA , Chung I , Whitworth AJ , Hirst J . Cryo‐EM structures of mitochondrial respiratory complex I from Drosophila melanogaster. eLife. 2023;12:e84424. 36622099 10.7554/eLife.84424 PMC9977279

[109] Cheng H , Deumens E , Freericks JK , Li C , Sanders BA . Application of quantum computing to biochemical systems: a look to the future. Front Chem. 2020;8:587693. 10.3389/fchem.2020.587143 PMC7732423 33330375

[110] Moe A , Di Trani J , Rubinstein JL , Brzezinski P . Cryo‐EM structure and kinetics reveal electron transfer by 2D diffusion of cytochrome c in the yeast III‐IV respiratory supercomplex. Proc Natl Acad Sci USA. 2021;118(11):e2022310118. 33836592 10.1073/pnas.2021157118 PMC7980474

[111] Parey K , Lasham J , Mills DJ , et al. High‐resolution structure and dynamics of mitochondrial complex I‐Insights into the proton pumping mechanism. Sci Adv. 2021;7(46):eabj3221. 34767441 10.1126/sciadv.abj3221 PMC8589321

[112] Matoušek M , Pernal K , Pavošević F , Veis L . Variational quantum eigensolver boosted by adiabatic connection. J Phys Chem A. 2024;128(3):687‐698. 38214999 10.1021/acs.jpca.3c07590 PMC10823474

[113] Doi H , Sugisaki K , Nakano T , Katagiri T , Mochizuki Y . Concurrent processing of VQE‐UCCSD calculations with the FMO scheme. Chem Lett. 2025;54(8):upaf145.

[114] Casares P , Campos R , and Martin‐Delgado M . QFold: quantum walks and deep learning to solve protein folding. Quantum Sci Technol. 2022;7(2):025013.

[115] Boulebnane S , Lucas X , Meyder A , Adaszewski S , Montanaro A . Peptide conformational sampling using the quantum approximate optimization algorithm. npj Quantum Inf. 2023;9(1):44.

[116] Cordier BA , Sawaya NPD , Guerreschi GG , McWeeney SK . Biology and medicine in the landscape of quantum advantages. J R Soc Interface. 2022;19(196):20220541. 36448288 10.1098/rsif.2022.0541 PMC9709576

[117] Huang R , Li C , Evangelista F , Leveraging small‐scale quantum computers with unitarily downfolded hamiltonians. PRX Quantum. 2023;4(2):020313.

[118] Gocho S , Nakamura H , Kanno S , et al. Excited state calculations using variational quantum eigensolver with spin‐restricted ansätze and automatically‐adjusted constraints. npj Comput Mater. 2023;9(1):85.

[119] Chin AW , Prior J , Rosenbach R , Caycedo‐Soler F , Huelga SF , Plenio MB . The role of non‐equilibrium vibrational structures in electronic coherence and recoherence in pigment–protein complexes. Nat Phys. 2013;9(2):113‐118.

[120] Fan S , Gao H , Zhang Y , et al. Quantum sensing of free radical generation in mitochondria of single heart muscle cells during hypoxia and reoxygenation. ACS Nano. 2024;18(4):2982‐2991. 38235677 10.1021/acsnano.3c07959 PMC10832053

[121] Lin N , van Zomeren K , van Veen T , et al. Quantum sensing of free radicals in primary human granulosa cells with nanoscale resolution. ACS Cent Sci. 2023;9(9):1784‐1798. 37780363 10.1021/acscentsci.3c00747 PMC10540281

[122] Matarèse B , Rusin A , Seymour C , Mothersill C . Quantum biology and the potential role of entanglement and tunneling in non‐targeted effects of ionizing radiation: a review and proposed model. Int J Mol Sci. 2023;24(22):16464. 38003655 10.3390/ijms242216464 PMC10671017

[123] Nunn AV , Guy GW , Bell JD . The quantum mitochondrion and optimal health. Biochem Soc Trans. 2016;44(4):1101‐1110. 27528758 10.1042/BST20160096 PMC5264502

[124] Ghamsari M , Baniasadi F . Quantum for biology: spectroscopy and sensing. Innov Emerg Technol. 2025;11:2430005.

[125] Wiest M . A quantum microtubule substrate of consciousness is experimentally supported and solves the binding and epiphenomenalism problems. Neurosci Conscious. 2025;2025(1):niaf011. 40342554 10.1093/nc/niaf011 PMC12060853

[126] Hameroff S , Penrose R . Orchestrated reduction of quantum coherence in brain microtubules: a model for consciousness. Math Comput Simul. 1996;40(3‐4):453‐480.

[127] Hameroff S . How quantum brain biology can rescue conscious free will. Front Integr Neurosci. 2012;6:93. 23091452 10.3389/fnint.2012.00093 PMC3470100

[128] Barjas Qaswal A . Quantum tunneling of ions through the closed voltage‐gated channels of the biological membrane: a mathematical model and implications. Quantum Rep. 2019;1(2):219‐225.

[129] Salari V , Naeij H , Shafiee A . Quantum interference and selectivity through biological ion channels. Sci Rep. 2017;7(1):41627. 28134331 10.1038/srep41625 PMC5278555

[130] Çelebi G , Özçelik E , Vardar E , Demir D . Time delay during the proton tunneling in the base pairs of the DNA double helix. Prog Biophys Mol Biol. 2021;167:96‐103. 34118266 10.1016/j.pbiomolbio.2021.06.001

[131] Chandra A , Aswal D , Need of quantum biology to investigate beneficial effects at low doses (< 100 mSv) and maximize peaceful applications of nuclear energy. Mapan. 2023;39(1):5‐24.

[132] Kinsey L , Beane W , Tseng K . Accelerating an integrative view of quantum biology. Front Physiol. 2024;14:1349990. 10.3389/fphys.2023.1349013 PMC10811782 38283282

[133] Chopra D . Towards quantum medicine. J Altern Complement Integr Med. 2022. 8(3):1‐14.

[134] Yun H , Lee S , Kim H , et al. Advancing biosensing and bioimaging with quantum technologies: from fundamental science to medical applications. Appl Phys Rev. 2025;12(3):031301.

[135] Chin A , Huelga S , Plenio M . Coherence and decoherence in biological systems: principles of noise‐assisted transport and the origin of long‐lived coherences. Philos Trans R Soc A: Math Phys Eng Sci. 2012;370(1972):3638‐3657. 10.1098/rsta.2011.0224 22753818

[136] Dorner R , Goold J , Vedral V . Towards quantum simulations of biological information flow. Interface Focus. 2012;2(4):522‐528. 23919131 10.1098/rsfs.2011.0109 PMC3363031

[137] Fauseweh B . Quantum many‐body simulations on digital quantum computers: state‐of‐the‐art and future challenges. Nat Commun. 2024;15(1):8015. 38459040 10.1038/s41467-024-46402-9 PMC10923891

[138] Jedlicka P . Revisiting the quantum brain hypothesis: toward quantum (Neuro)biology? Front Mol Neurosci. 2017;10:366. 29163041 10.3389/fnmol.2017.00366 PMC5681944

[139] Scholes G , Fleming G . What is quantum biology? Proc Natl Acad Sci USA. 2026;123(14):e2531134123. 41860951 10.1073/pnas.2531134123 PMC13056094

[140] Usselman RJ , Chavarriaga C , Castello PR , et al. The quantum biology of reactive oxygen species partitioning impacts cellular bioenergetics. Sci Rep. 2016;6:38543. 27995996 10.1038/srep38543 PMC5172244

[141] Rinaldi A . When life gets physical. EMBO Rep. 2011;13(1):24‐27. 22173031 10.1038/embor.2011.236 PMC3246260

[142] Mokhtari M , Khoshbakht S , Ziyaei K , Akbari ME , Moravveji SS . New classifications for quantum bioinformatics: q‐bioinformatics, QCt‐bioinformatics, QCg‐bioinformatics, and QCr‐bioinformatics. Brief Bioinform. 2024. 25(2):bbae044. 38446742 10.1093/bib/bbae074 PMC10939336

